# Evaluation of a Complex Intervention to Strengthen Participation-Centred Care for Children with Special Healthcare Needs: Protocol of the Stepped Wedge Cluster Randomised PART-CHILD Trial

**DOI:** 10.3390/ijerph192416865

**Published:** 2022-12-15

**Authors:** Michael Eichinger, Tatiana Görig, Sabine Georg, Dorle Hoffmann, Diana Sonntag, Heike Philippi, Jochem König, Michael S. Urschitz, Freia De Bock

**Affiliations:** 1 Center for Preventive Medicine and Digital Health, Medical Faculty Mannheim, Heidelberg University, 68167 Mannheim, Germany; 2 Division of Pediatric Epidemiology, Institute of Medical Biostatistics, Epidemiology, and Informatics, University Medical Center of the Johannes Gutenberg-University Mainz, 55131 Mainz, Germany; 3 Department of Medical Informatics, Biometry and Epidemiology, Friedrich-Alexander-Universität Erlangen-Nürnberg, 91054 Erlangen, Germany; 4 Social Pediatric Centre Frankfurt, 60316 Frankfurt, Germany; 5 Department of General Pediatrics, Neonatology and Pediatric Cardiology, Medical Faculty, University Hospital Düsseldorf, Heinrich-Heine-University, 40225 Düsseldorf, Germany

**Keywords:** child, International Classification of Functioning, Disability and Health, participation-centred care, shared decision making, participation, special healthcare needs

## Abstract

Introduction: Participation is an important dimension of healthy child development and is associated with higher self-rated health, educational attainment and civic engagement. Many children with special healthcare needs (SHCN) experience limited participation and are thus at risk for adverse health and developmental outcomes. Despite this, interventions that promote participation in healthcare are scarce. We therefore evaluate the effectiveness of a complex age- and condition-generic intervention that strengthens participation-centred care involving parents and their children with SHCN by, *inter alia*, assessing preferences, specifying participation goals and facilitating shared decision-making in care. Methods and analysis: In this study protocol we describe the design and procedures for an unblinded, stepped wedge, cluster randomised trial conducted in 15 German interdisciplinary healthcare facilities providing services for children aged 0–18 years with SHCN. Sites are randomised to five periods in which they switch from control to intervention condition in blocks of three. The intervention includes: (1) team training focused on participation-centred care, (2) introduction of a new software facilitating participation-focused documentation and (3) implementation support promoting the transfer of training content into routine care. Study sites deliver routine care while in the control condition. As primary outcome, the degree of perceived shared decision-making with parents (CollaboRATE^pediatric^ parent scale), a potential antecedent of achieving participation goals in everyday life, is assessed on one randomly selected day per week during the entire study period, directly following care appointments. We aim to sample 70 parents per study site and period. Additionally, participation of children is assessed within a closed embedded cohort with three parent and patient surveys. Intervention effectiveness will be modelled with a marginal model for correlated binary outcomes using generalised estimation equations and complete cases. A comprehensive mixed-methods process evaluation complements the effectiveness analyses.

## 1. Introduction

Participation, broadly defined here as a person’s “involvement in life situations”, is considered an important phenomenon thought to be a prerequisite for children to realise their full potential as members of society. It represents one of the key components of the International Classification of Functioning, Disability and Health–Child and Youth Version (ICF-CY) in addition to body functions/structures and activities [1,2]. Previous work suggests that participation is associated with higher self-rated health, life satisfaction, wellbeing, perceived academic performance and civic engagement [3,4]. Moreover, health policy both nationally and internationally increasingly emphasises participation as an important objective of paediatric healthcare services [5] and international human rights treaties such as the Convention on the Rights of Persons with Disabilities or the Convention on the Rights of the Child demand that children with disabilities can and should fully and effectively participate in society and have access to services promoting participation [6,7].

Children and adolescents (hereinafter referred to as *children*) with special healthcare needs (SHCN) often experience lower levels of participation compared to their healthy peers [8,9,10,11,12]. Participation can be limited in different life domains including interpersonal relationships [11,12], recreational activities [8,12] or community life [10]. Although reduced levels of participation are common in children with SHCN, the delivery of healthcare services is largely focused on reducing physical impairments, dysfunctions and symptoms, with comparatively less emphasis on participation as an objective of service delivery. Broadening the scope of service delivery to include participation as an important additional objective and outcome has therefore been called for [13,14].

**Figure 1:**
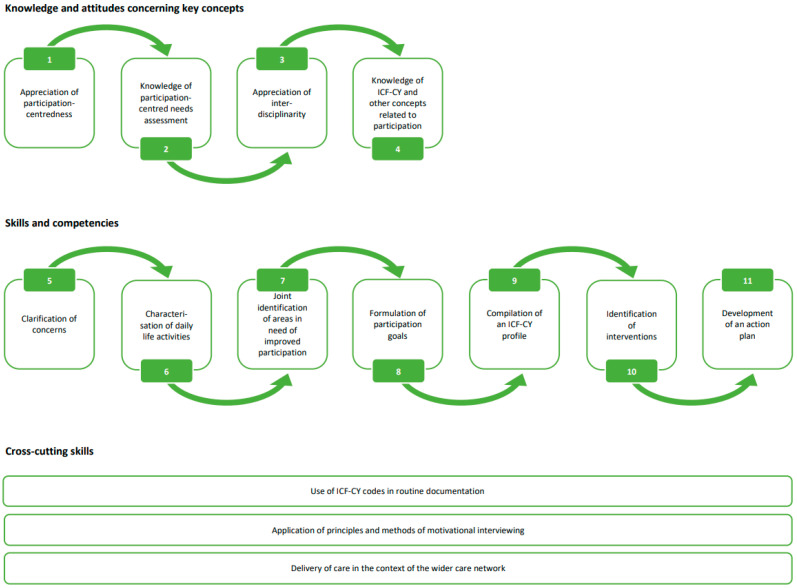
Framework for participation-centred care. ICF-CY, International Classification of Functioning Disability and Health–Child and Youth Version.

Against this backdrop, we examine participation-centred care, defined as service delivery that recognises participation in all ICF-CY life domains as an important *outcome of healthcare* and, to this end, elicits patient participation and preferences in *care processes*. Overlapping to some extent with the more widely used concept of patient-centred care, which generally deals with information sharing and patient involvement [15], participation-centred care is more narrowly focused on promoting participation, both in the sense of involvement in the processes of care (i.e., in decision-making) and with the goal of increasing involvement in everyday life. Our clinical experience suggests that participation-centred care objectives that are meaningful and relevant to patients and parents are a promising avenue for improving service delivery to children with SHCN in whom cure as a care objective is not attainable. The work presented here is based on a framework for fostering participation-centred care that emanated from practical care experience for children with SHCN and from development and feasibility work in six Social Pediatric Centers (Figure 1). It comprises aspects of both shared decision making (SDM) and the ICF-CY. SDM, i.e., *involvement in the process of care*, is a potential antecedent and enabler of participation, *an aim and potential outcome of care*. SDM thus has the potential to contribute to and enable participation-centred care, specifically when determining participation needs and goals, in three domains: (1) the provision of information on health issues, diagnostic and treatment options potentially affecting participation, (2) the elicitation of child and parent preferences and (3) the integration of these preferences into healthcare decisions [16,17]. In total, seven out of 14 elements of the framework for participation-centred care directly contribute to improved SDM (elements 1, 2, 6, 7, 8, 11, 13 in Figure 1), highlighting the importance of SDM as an enabler of participation-centredness and ultimately participation in everyday life. Eliciting participation needs and goals from children and their parents can be supported by applying principles and procedures of motivational interviewing [18]. However, successfully implementing SDM in routine care is challenging. Most decisions in paediatric healthcare are still taken by healthcare professionals with children presumably being less frequently involved in decisions compared to their parents [19,20,21]. As a complementary component, the ICF-CY [2] has potential to provide a common framework and language for interdisciplinary care teams, children and parents to jointly identify participation goals. Despite this potential, however, efforts to implement the ICF-CY in routine care have been scarce with evidence on effectiveness lacking [22].

The framework for participation-centred care addresses these implementation challenges as it is aimed at positively influencing health care provider knowledge, attitudes, skills and competencies (Figure 1). Summarised briefly, the framework consists of 14 elements divided into three sections. The first section (elements 1–4) comprises fundamental values, attitudes and basic knowledge of concepts related to participation including SDM, contributing to the determination of participation needs, preferences and goals (steps 7 and 8 of the intervention framework), and the ICF-CY. The second section (elements 5–11) focuses on specific skills and competencies needed to align service delivery with participation-centred care and serves as a blueprint for structuring care processes with children and their parents in routine care. Whilst the skills and competencies are arranged linearly for ease of presentation, healthcare professionals often use elements of the framework iteratively in complex care processes. The third section encompasses cross-cutting skills that contribute to the successful implementation of elements in Section 2. A detailed description of the framework is presented in Online Supplementary File S6.

A recent systematic review suggested that interventions primarily targeting body functions or activity performance had little effect on participation while those directly targeting participation by providing children and parents with individual coaching sessions, setting mutually agreed-upon participation goals and selecting the most appropriate therapeutic activities for achieving the participation goals [23,24] demonstrated greater effects [25]. To date, interventions have targeted narrowly defined subgroups of children with SHCN such as those in foster care or those with social difficulties [23,26] and have been conducted by single professional groups (e.g., educators, occupational therapists or psychologists) [23,24,26]. However, approaches that are condition- and potentially age-generic may be particularly useful because service delivery processes might be comparable across different chronic conditions [27] and care fragmentation can simultaneously be counteracted. Given that care for children with SHCN almost always involves healthcare professionals from several different disciplines and institutions, interventions that target entire care teams or interdisciplinary care networks might be more effective than interventions involving single professional groups. Moreover, our understanding of the pathways through which intervention have effect and factors enabling or hindering their implementation remains limited. Yet, this knowledge could be used to improve existing interventions by identifying and replacing less effective intervention components.

### Objectives

We describe the study design for implementing and evaluating a complex age- and condition-generic intervention to strengthen the delivery of participation-centred care aimed at all healthcare providers in interdisciplinary outpatient clinics for children with SHCN primarily affected by neurological, neurodevelopmental and behavioural chronic health conditions and disabilities. Specifically, we describe the methods used to

(1) assess the effectiveness of the PART-CHILD intervention to improve perceived SDM with parents (primary endpoint) and explore effectiveness in a variety of secondary endpoints (e.g., participation) related to the framework for participation-centred care (*effectiveness evaluation*) and
(2) explore (a) implementation of the PART-CHILD intervention, (b) its mechanisms of impact and (c) contextual factors potentially moderating intervention effectiveness (*process evaluation*).

## 2. Intervention

**Figure 2:**
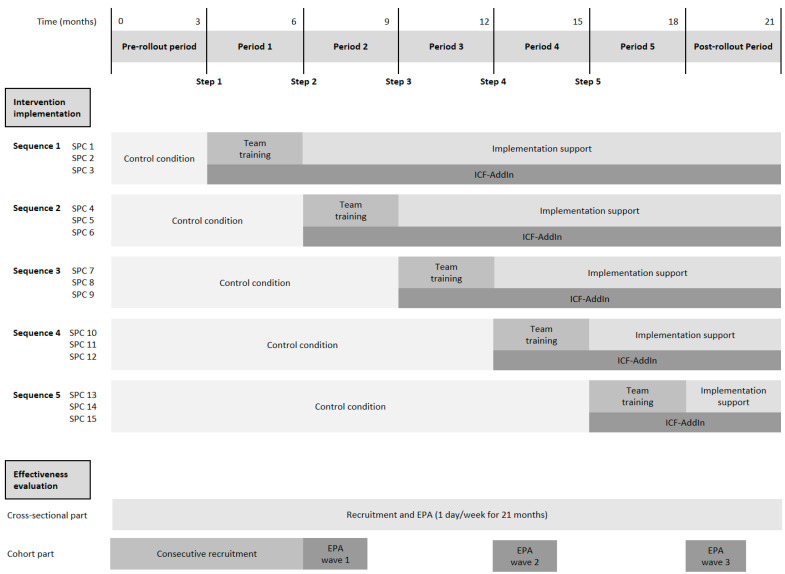
Design of the effectiveness evaluation. For SPC 13–15 the implementation support is continued beyond the end of the effectiveness evaluation (not shown in the figure). EPA, endpoint assessment; SPC, social paediatric centre.

The PART-CHILD intervention targets entire care teams at the study sites and comprises three modules described briefly below. We anticipate that the modules interact with each other in complex ways, potentially increasing intervention effectiveness over and above the effectiveness of a one-component intervention. For example, the provision of the software *ICF-AddIn* might improve the ICF-CY-based documentation of participation goals covered in the team training and the implementation support program and might thus strengthen the long-term maintenance of team training content in routine care. The intervention modules are sequentially implemented across study sites (Figure 2).

### 2.1. Module 1: Team Training

All staff members providing diagnostic, therapeutic and counselling services including the SPC director participate in team training based on the framework for participation-centred care (Figure 1). Training is delivered in two blocks over two days each within a three-month period, with the first block introducing staff to the ICF-CY [2], its use, concepts related to participation such as the biopsychosocial model of health [28] and participation-centred needs assessment. The second block features hands-on activities for formulating participation goals and action plans with children and parents and developing communication skills based on motivational interviewing [18]. Team training is also intended to provide space for discussions on how training content could be sustainably transferred into routine use and to promote development of a common language and an institution-wide, shared understanding of participation-centred care.

The team training, and elements for supporting implementation (module 3, described below), are based on detailed manuals, shared train-the-trainer experiences and a standardised set of presentation slides and training materials to ensure comparable intervention delivery across study sites. Training takes place on site, delivered by interdisciplinary pairs of experienced trainers actively practising in SPCs. To harness complementary knowledge and practical experience and to parallel the interdisciplinary teamwork and differing roles within SPCs necessary for formulating participation goals and action plans according to the ICF-CY, pairs in the first block consist of a board-certified paediatrician and an allied health professional (e.g., physiotherapist or occupational therapist). Pairs in the second block include an allied health professional and an experienced trainer in motivational interviewing.

### 2.2. Module 2: Documentation Software ICF-AddIn

Based on the ICF-CY, the *ICF-AddIn* software facilitates documentation of care processes using an innovative everyday language keyword search function, exports ICF-CY codes to different types of clinical documents such as notes or discharge letters and enables involvement of children and parents in the joint documentation of action plans resulting from consultations. To ease use and access, the software is incorporated into the existing medical information systems prior to the team training.

### 2.3. Module 3: Implementation Support

The implementation support program aims to empower SPC directors and their staff to transfer training content into routine care. In contrast to the highly standardised modules 1 and 2, the implementation support program is tailored to the local needs of each SPC to maximise intervention effectiveness [29]. Following team training, the implementation team leader conducts a 1–2 h semi-structured needs assessment with the SPC director to identify the desired elements of the intervention support program. After the needs assessment, the SPC director chooses different support elements from a predefined menu (Table 1). The elements of the implementation support are aligned with the Expert Recommendations for Implementing Change (ERIC), an international consensus-based compilation of implementation strategies [30]. The most relevant ERIC implementation strategies for each element of the implementation support program are reported in the second column of Table 1. All elements are implemented over six months following team training by the same pool of trainers that implement module 1, with timing based on study sites’ needs.

## 3. Methods and Analysis

### 3.1. Study Design

The study design reflects the UK Medical Research Council guidance for the assessment of complex interventions [31] and comprises two parts. The effectiveness evaluation is based on a stepped wedge cluster randomised trial (Figure 2) with all study sites receiving the intervention at one of five randomly allocated time points. After a three-month pre-rollout period, three study sites cross from the control to the intervention condition every three months. Together with a three-month post-rollout period after the last study sites receive the intervention, the total length of the effectiveness evaluation is 21 months. The process evaluation uses a convergent mixed-methods design [32] to assess the implementation process of the PART-CHILD intervention, to uncover mechanisms potentially leading to intervention effects and to explore contextual factors that may moderate intervention effectiveness.

### 3.2. Reporting

The reporting in this study protocol is based on the extension of the CONSORT 2010 statement for reporting stepped wedge cluster randomised trials [33] and the SPIRIT statement for reporting clinical trial protocols [34]. The completed SPIRIT Checklist is provided in Online Supplementary File S4.

### 3.3. Ethical Considerations

The primary ethics approval was obtained from the Medical Ethics Review Board of the Medical Faculty Mannheim at Heidelberg University (2018-529N-MA). Secondary approvals were granted for all study sites by their respective ethics review boards. Written informed consent to participate in the study is obtained from all parents, patients and staff members providing pseudonymous data in the cohort component of the effectiveness evaluation as well as the process evaluation. The consent forms are provided in Online Supplementary File S5. The cross-sectional part of the effectiveness evaluation is exempt from written informed consent as only anonymous data are collected.

### 3.4. Setting

The intervention is implemented in 15 Social Paediatric Centres (SPCs) across Germany. SPCs are secondary and tertiary healthcare facilities providing diagnostic, counseling and treatment services for children aged 0–18 years with special healthcare needs or suspected to have special healthcare needs. This includes, but is not limited to children with neurological, neurodevelopmental and behavioural chronic health conditions and disabilities (e.g., epilepsy, cerebral palsy or attention deficit hyperactivity disorder). The 156 German SPCs are interdisciplinary, physician-led outpatient clinics consisting of paediatricians (often with sub-specialty training in neuropaediatrics), psychologists, physiotherapists, occupational therapists, other allied health professionals like nurses or music therapists, and social workers.

SPCs vary considerably in terms of size and the range of services provided. For example; more than two-thirds of German SPCs provide specialised interdisciplinary diagnostic work-ups only (henceforth *diagnostic SPCs*) with recommended therapeutic services provided by other healthcare providers. The remaining SPCs provide both diagnostic work-ups and in-house therapeutic services (henceforth *diagnostic and treatment SPCs*).

### 3.5. Inclusion and Exclusion Criteria for Study Sites

Eligibility of study sites was defined by: (1) consent of the SPC director to participate in all components of the intervention along with the entire staff, (2) availability of a venue to host the PART-CHILD team training, (3) use of an electronic medical information system, and (4) provision of information technology (IT) resources to facilitate roll-out of the documentation software *ICF-AddIn*. Exclusion criteria included (1) the existence of state laws or regulations of local data protection boards prohibiting use of anonymised data from medical information systems for research purposes, (2) SPC staff previously exposed to ICF-CY-based training programs, (3) foreseeable changes in SPC leadership, institutional affiliation or the IT infrastructure during the study period and (4) current or planned negotiations with health insurance funds that might impact SPC operation.

### 3.6. Recruitment and Selection of Study Sites

**Figure 3:**
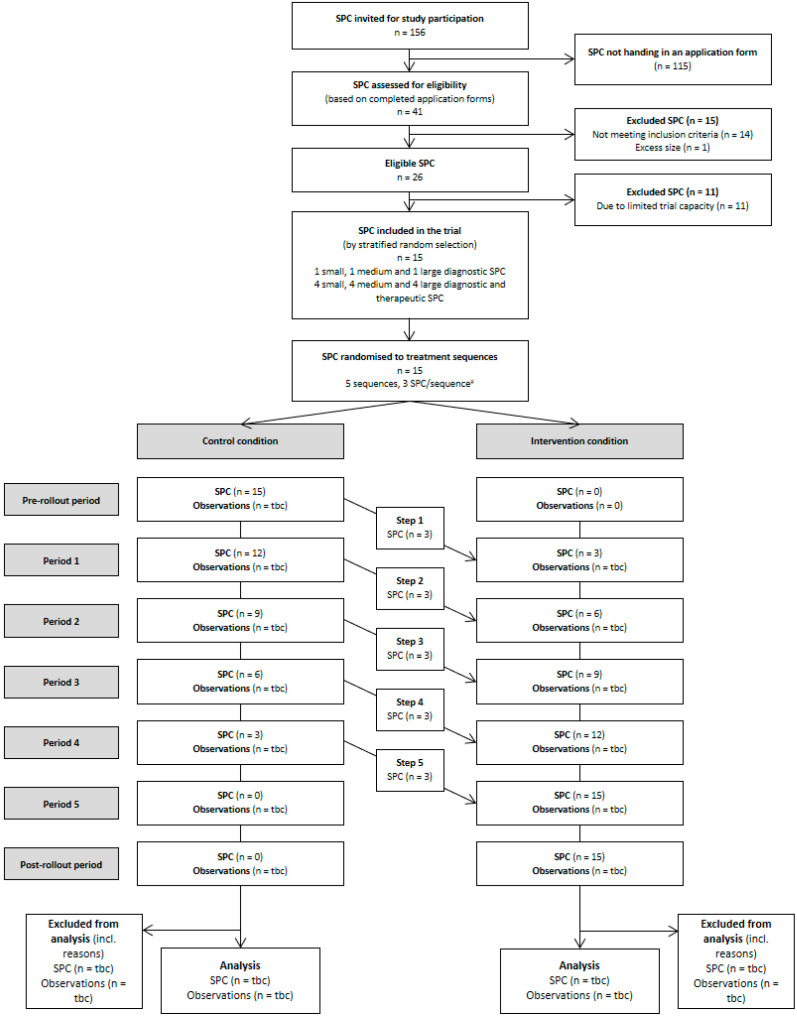
CONSORT flowchart for the primary endpoint. ^#^ After randomisation, but prior to the start of data collection, one medium-sized diagnostic SPC withdrew due to unforeseen circumstances during the study period. This SPC was replaced by a randomly selected study site belonging to the same stratum. SPC, social paediatric centre; tbc, to be completed after completion of data collection.

Figure 3 provides an overview of the recruitment and selection process of study sites. In March 2018, we used the mailing lists of two national German SPC associations and a presentation at the annual meeting of German SPCs to invite applications for study participation. By 30 April 2018, 41 of 156 SPCs (26%) submitted a completed application with 27 (66%) meeting all inclusion and no exclusion criteria (described above). We excluded one eligible study site from consideration that had an unusually large patient volume and a staff size (n = 190) that would have both resulted in unbalanced data collection across study sites and exceeded the training capacity of the study team.

Based on prior sample size calculations reported below, the trial statistician selected a random sample of fifteen SPCs from the 26 eligible study sites in June 2018, stratified by patient volume (small [1700–2600 referrals per 3 months], medium [3870–4934 referrals], large [5000–12,027 referrals]) and the scope of services (diagnostic versus diagnostic and treatment SPCs). The final set of study sites included three diagnostic SPCs (one of each size) and twelve diagnostic and therapeutic SPCs (four of each size). In August 2018, one medium-sized diagnostic SPC withdrew due to unforeseen circumstances and was replaced by the trial statistician, who randomly selected another site from eligible SPCs belonging to the same stratum.

### 3.7. Logic Model

**Figure 4:**
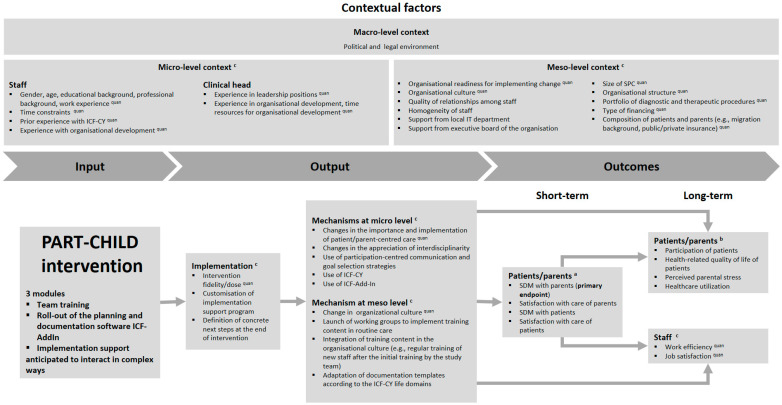
Logic model of the PART-CHILD intervention. We distinguish between (1) contextual factors, (2) inputs, (i.e., the components of the PART-CHILD intervention), (3) outputs and (4) both short- and long-term outcomes. These aspects are covered in the quantitative ^a^ cross-sectional and ^b^ cohort component of the effectiveness evaluation and the qualitative and ^quan^ quantitative ^c^ process evaluation. Macro-level contextual factors are not covered in the evaluation, but are considered when interpreting the findings of the trial. ICF-CY, International Classification of Functioning Disability and Health–Child and Youth Version; SDM, shared decision making; SPC, social paediatric centre.

The effectiveness and process evaluation are based on a logic model summarising potential endpoints and mechanisms of impact of the PART-CHILD intervention targeted in the evaluation (Figure 4). Following widely used guidance [35], we distinguish between (1) contextual factors, (2) inputs, i.e., the components of the PART-CHILD intervention anticipated to interact in complex ways potentially increasing intervention effectiveness above and beyond the effectiveness of a one-component intervention, (3) outputs and (4) both short- and long-term outcomes. Contextual factors constitute the setting in which the intervention is implemented and have the potential to moderate intervention effects [36]. We differentiate between contextual factors at the micro (i.e., staff), meso (i.e., SPC) and macro level (i.e., the political and legal environment). Whilst macro-level contextual factors are not included in the evaluation, they are considered when interpreting the findings of the trial.

We hypothesise that the intervention effects are mediated by the quality of the intervention implementation and mechanisms of impact at the micro level, such as the use of participation-centred communication strategies by staff, and the meso level, such as changes in organisational culture and processes. Outputs in turn affect short-term outcomes in children and parents (e.g., perceived SDM, satisfaction with care) and influence long-term outcomes (e.g., participation of children, job satisfaction among staff) directly or indirectly through short-term outcomes (Figure 4).

Given that the intervention components are anticipated to interact in complex ways, potentially increasing intervention effectiveness over and above the effectiveness of a one-component intervention, it is not possible to map mechanisms of impact and outcomes to specific intervention components. For example, the team training, the implementation of the *ICF-AddIn* and several components of the implementation support all facilitate the use of the ICF-CY, a potential mechanism of impact at the micro level (Figure 4). Mechanisms of impact and outcomes thus reflect the combined effect of all intervention components.

### 3.8. Effectiveness Evaluation

#### 3.8.1. Evaluation Design

The effectiveness evaluation has two parts. A cross sectional component uses data from contacts between children, parents and SPC staff to assess the primary endpoint -changes in SDM -within and across study sites and to explore change in several secondary endpoints at the parent- and patient-level. A cohort component uses an embedded closed cohort design to explore changes in participation and quality of life of children with SHCN.

#### 3.8.2. Randomisation

Following stratified randomisation in August 2018, study sites were assigned to one of five sequences. First diagnostic SPCs were sorted in ascending order by size and then randomly assigned to sequence 1, 3 and 5. SPCs providing both diagnostic and treatment services were then distributed across the sequences such that each sequence contained three centres of different size (i.e., one small, medium and large SPC).

#### 3.8.3. Allocation Concealment and Blinding

We intended to keep the study sites’ allocation to treatment sequences concealed until formal cooperation agreements with each site had been signed. However, this goal was not attained due to delays in finalising cooperation agreements. No SPC withdrew from the study after the sequence had been disclosed. Given its design involving the unidirectional crossing of study sites from the control to the intervention condition, it is not possible to blind SPC staff, children and their parents, the implementation team delivering the intervention or the study statistician.

#### 3.8.4. Sample Size Considerations

We assume one half of the intervention effect to be present in the period when the respective study site crosses to the intervention condition and the full intervention effect to be present for all subsequent periods. SDM with parents, the primary endpoint, is assessed in the cross-sectional component using the binary CollaboRATE^pediatric^ score that differentiates between suboptimal and optimal extents of perceived SDM [37]. Assuming a time constant cluster effect with standard deviation 0.06 of the cluster specific proportions of responses indicating an optimal extent of SDM, a practically relevant difference between 0.60 and 0.675 can be detected with a two-sided test with type 1 error of 0.05 and a power of 0.80, if 61 parents are sampled in each SPC in each quarter. Allowing for 10% incomplete questionnaires, we aim to sample 70 parents per study site and quarter. Sensitivity analyses relaxing simplifying assumptions (e.g., constant sample sizes across periods and study sites, no time varying cluster effects) did not affect the power calculations substantially. We expect that the proportion of parents providing data on more than one occasion will be very small, so this will be ignored for purposes of power calculation and in the analyses.

#### 3.8.5. Participants

##### Cross-Sectional Component

We invite all parents having an appointment at one of the SPCs to participate in this component of the evaluation without applying any further inclusion or exclusion criteria. Children are invited to participate starting at age seven years (grade 2 of primary school) when most children have acquired sufficient reading and writing competencies to complete a short questionnaire. However, parents and children lacking German language skills that would allow them to complete a short questionnaire or children with severe cognitive disabilities (assessed by parent reports) are excluded *de facto*.

##### Cohort Component

Parents are eligible for participation if (1) they are able to complete a questionnaire in German, (2) their child is aged 3–16 years and (3) they are expected to have at least four more appointments at the SPC over the following two years. Children of participating parents aged seven years and older with sufficient German language skills and without severe cognitive disabilities (based on parental assessment) are also invited to participate in this part of the evaluation. We exclude parents with children aged below 3 years because there is no conceptual clarity on involvement in life situations in this age group and valid instruments for assessing participation are not available [38].

#### 3.8.6. Recruitment and Data Collection

##### Cross-Sectional Component

Data are collected on one randomly selected day per week during the entire study duration. To prevent the pre-selection of participants in the evaluation, study sites are notified no more than eight days in advance about the next data collection day. The study statistician used a block randomization scheme such that three Mondays to Wednesdays and two Thursdays and Fridays were randomly selected per quarter. Upon registration, receptionists invite all parents and children seven years and older to participate. After the appointment, parents and older children are reminded about the questionnaires and are asked to complete them in the reception area. Completed questionnaires are deposited in a locked box at each study site. In general, participants complete the survey only once. While we cannot rule out that a small number of families take part multiple times, this is quite unlikely as most families have appointments at the SPC only every couple of weeks and data is collected on one random day per week. Given logistical constraints at several study sites including lack of internet access, it is not feasible to provide participants with an option to complete an online survey *in lieu* of a paper-and-pencil questionnaire. While completing an online survey is a convenient way of participation for a subset of parents, we do not anticipate that the mode of administration will induce selection bias.

##### Cohort Component

SPC staff recruit eligible parents and children consecutively at the end of their appointment. After informing the families about the study, staff obtain written informed consent from parents and children.

The closed cohort comprises three parent and child surveys completed once every six months. Depending on their preferences, families complete paper-and-pencil or online surveys implemented in Limesurvey (Version 2.65.0, LimeSurvey GmbH, Hamburg, Germany). Completed surveys are either returned by mail using prepaid reply envelopes or are submitted online. To minimise loss to follow-up, participants receive up to three reminders by mail or email.

#### 3.8.7. Endpoints

We use previously validated instruments to assess the primary and all secondary endpoints. All endpoints, their method of operationalisation and references to validation studies are summarised in Table 2. Secondary endpoints assessed in the cohort component are included in all three surveys.

#### 3.8.8. Statistical Analyses

To assess the intervention effect on perceived SDM with parents (primary endpoint; binary variable: optimal versus suboptimal perceived SDM) we will fit a marginal model for correlated binary data using generalised estimation equations and complete cases. The model will be applied to data aggregated at the level of study sites and study periods and will comprise fixed effects for study period and an intervention indicator. The intervention indicator will be set to 0, 0.5 and 1 for control, the first intervention and all subsequent intervention periods, respectively. For the sequence of eight random period effects per SPC, we specified a compound symmetry correlation structure with homogeneous variances. The Mancl and DeRouen method will be used to account for inflation of standard errors and type one errors [44]. The intervention effects will be reported as odds ratios with 95% confidence intervals contrasting the intervention condition to the control condition. The SAS Code for fitting the model for the primary endpoint is provided in Online Supplementary File S1. We will apply equivalent modeling strategies for the other binary endpoints in the cross-sectional component. For continuous endpoints in the cross-sectional component, we will fit linear mixed models with fixed effects for study period and intervention indicator and random effects at the SPC and SPC-period level using robust variance estimation.

For continuous endpoints in the cohort component, we will fit linear mixed models using survey wave as a discrete time variable. The models will comprise random intercepts at SPC, SPC-survey and subject levels, respectively, and fixed effects for survey wave, intervention indicator and age. The intervention indicator will be defined as above. When applicable, we will use robust standard errors and Wald tests based on sandwich estimators for effect estimation.

### 3.9. Process Evaluation

**Figure 5:**
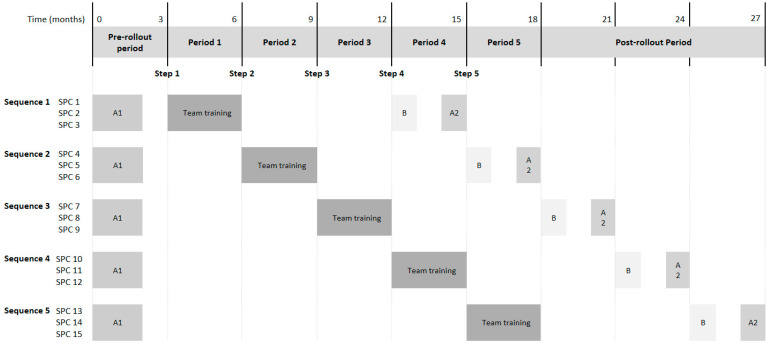
Overview of the process evaluation. We conduct baseline (A1) and follow-up surveys (A2) and semi-structured telephone interviews (B) with SPC directors and staff. SPC, social paediatric centre.

Following existing guidance [36], we will (1) analyse intervention implementation using the RE-AIM framework [45], (2) explore mechanisms potentially explaining intervention effects and (3) investigate contextual factors that may act as barriers or facilitators to intervention implementation and effectiveness (Figure 5). We use a convergent mixed methods approach integrating qualitative and quantitative data [32]. Specifically, the database to which RE-AIM will be applied derives from baseline (A1 in Figure 5) and follow-up surveys (A2) and semi-structured telephone interviews with SPC directors and their staff (B) as well as comprehensive documentation of the intervention process based on implementation notes, *ICF-AddIn* usage and consultation observations.

#### 3.9.1. Participant Recruitment and Data Collection

##### Surveys among SPC Directors and Their Staff

We invite all eligible participants for team training to take part in two surveys administered at baseline prior to intervention implementation and at follow-up on a rolling basis approximately nine months after the team training (Figure 5). Following initial contact about study design and objectives, the SPC directors distribute written study information, the consent form and the paper-and-pencil baseline questionnaire to staff members, who are then asked to return completed documents in individually sealed envelopes to ensure confidentiality.

##### Semi-Structured Interviews with SPC Directors and Their Staff

The SPC directors of study sites are asked to suggest four staff members of their staff thought to have diverse perspectives (e.g., different professional groups, attitudes towards and experiences with participation-centred care). The study team randomly selects two of the four candidates and obtains written informed consent. Semi-structured interviews are conducted with the 15 SPC directors and 30 staff members (2 per SPC) approximately six months after the team training at the respective study sites by two members of the research team with expertise in qualitative research methods (TG: PhD, female, sociologist; SG: MA, female, educational scientist). All interviews are audio-recorded and transcribed verbatim.

#### 3.9.2. Outcomes

##### Surveys among SPC Directors and Their Staff

As outlined in the description of our logic model, the baseline survey assesses preconditions for intervention implementation including (1) contextual factors (e.g., organisational readiness for change [validated scale: ORIC] [46], organisational culture [Competing Values Instrument] [47], perceived importance and feasibility of implementing patient- and parent-centred care [Patient involvement scale] [48]), (2) staff characteristics (e.g., professional backgrounds, level of awareness of the ICF-CY, perceived work satisfaction [Global Job Satisfaction Scale] [49]) and (3) patient characteristics (e.g., migration background, health insurance status).

The follow-up survey investigates changes in care practices and pathways to change using Likert scales and open-ended questions including (1) the implementation of training content and the use of the *ICF-AddIn* in daily routine care, (2) satisfaction with the intervention, (3) changes in perceived importance and feasibility of patient- and parent-centred care (Patient involvement scale) [48], (4) changes in perceived work satisfaction (Global Job Satisfaction Scale) [49] and (5) perceived barriers for the implementation of training content. Items for assessing some staff and patient characteristics were developed specifically for use in this study and have not been previously tested. The surveys are provided in Online Supplementary File S2.

##### Semi-Structured Interviews with SPC Directors and Their Staff

Telephone interviews explore staff perspectives on the implementation of the intervention in routine care and contextual barriers and facilitators. Specifically, the interview guide covers the relevance and use of intervention components, satisfaction and post-intervention changes in staff attitudes towards participation-centred care. Interviews with SPC directors cover additional meso-level contextual factors (e.g., time or financial resources). The interview guides are provided in Online Supplementary File S3.

#### 3.9.3. Analysis

##### Surveys among SPC Directors and Their Staff

We will assess changes in the perceived importance and feasibility of implementing patient- and parent-centred care and perceived work satisfaction between baseline and follow-up. Descriptive analysis will assess satisfaction with intervention content and the degree to which training content is implemented and the *ICF-AddIn* is used. In addition, associations between contextual factors at the level of staff (micro-level) and SPCs (meso-level) (independent variables) with process measures such as implementation, adoption and reach will be analysed.

##### Semi-Structured Interviews with SPC Directors and Staff

Two members of the research team (TG and SG) independently code the interviews using MAXQDA (VERBI Software GmbH, Berlin, Germany) and apply qualitative content analysis according to Mayring [50], specifically paraphrasing coded text segments and subsequently summarising paraphrases with related meaning to analyse text material.

##### Implementation Notes, ICF-AddIn Usage and Consultation Observations

A comprehensive documentation of the intervention implementation including notes, and quantification of the ICF-AddIn usage will allow operationalization of RE-AIM components such as Reach (e.g., percentage of staff participating in the team training), Implementation of each of the three intervention components (e.g., number of components selected from the implementation support program, usage of ICF-AddIn), Adoption and Maintenance (e.g., formation of ICF-working groups at the study sites). Compliance of SPC staff with motivational interviewing principles during child-parent-staff consultations are monitored in a subsample [51].

### 3.10. Integration of Quantitative and Qualitative Data

To examine implementation processes, pathways to impact and differences across subgroups of SPCs (e.g., high versus low performing SPCs based on percentage reached across all RE-AIM parameters) [52], qualitative and quantitative data will be integrated in several ways at the analysis and interpretation stage. At the analysis stage, we will partly transform qualitative into quantitative data (e.g., number of actions an SPC implements to support the transfer of training content into routine care). The transformed data will, in turn, be used in graphical displays integrating information from both data types. In addition, the transformed data will feed into exploratory analyses to gain insight into intervention processes and potential differential implementation and effectiveness across study sites. At the interpretation stage, results of the semi-structured interviews will provide rich contextual information contributing to the interpretation of quantitative findings.

## 4. Discussion

The PART-CHILD study is one of the first studies to implement and evaluate a complex intervention to strengthen participation-centred care for children with SHCN [23,24]. Extending existing pilot approaches to implement electronic tools for participation-focused care planning [53] that uniquely center on participation as outcome, the PART-CHILD intervention intends to both facilitate participation of children and their parents in *processes of care* by strengthening SDM as well as participation as an *aim and potential outcome of care*. In the context of the proposed study, SDM is thus conceptualised as a potential antecedent and enabler of participation, comparable to previous participation-focused intervention studies [27]. In contrast to one-component interventions, the modules of the PART-CHILD intervention interact in complex ways potentially increasing effectiveness by mutually reinforcing their respective effects. For example, elements of the implementation support program such as advanced trainings in motivational interviewing or participation goal setting increase intervention dose and might thus improve medium-term intervention effectiveness and maintenance. The comprehensive process evaluation will help to discern how different intervention components interact and which combinations of components show particular potential to improve intervention effectiveness.

We anticipate this study will complement the small number of existing intervention studies in several ways. First, in contrast to the few published intervention studies focusing on single professions [23,24,26] or therapies [54], the PART-CHILD intervention deliberately embraces interdisciplinary care teams including paediatricians, nurses, allied health professionals and other non-medical staff. Targeting interdisciplinary care teams has the potential to strengthen interdisciplinary teamwork necessary to implement participation-centred care by promoting the development of an institution-wide shared understanding of participation-centredness and by accounting for the complementary roles of different professions in complex care processes for children with SHCN. Second, the PART-CHILD study has the potential to extend the existing evidence base from studies mainly targeting narrowly defined subgroups of children with SHCN [23,24] by taking a condition-generic approach. While highly specialised condition-specific care processes are essential for the diagnosis and treatment of children with rare diseases [55], a condition-generic approach might contribute to a coherent care framework for common paediatric conditions such as neurodevelopmental or behavioural disorders with often similar diagnostic work-ups and therapeutic strategies and thus help mitigate care fragmentation.

Third, whilst previous interventions attempt to improve the participation-centredness of interactions at the micro-level between professionals, children and their families [23,24], the PART-CHILD intervention acknowledges the complexities of implementing innovative interventions in existing healthcare institutions by targeting influences at the micro- and meso-levels. Especially the components targeting meso-level factors have the potential to improve long-term impact by supporting the adaptation of intervention components to increase the fit between the intervention and specific local contexts [56]. Last, as proposed for the field of speech-language pathology [27], we anticipate that the present study will provide first insights into the potential of the ICF-CY and SDM in achieving an institution-wide alignment of service delivery with participation goals of children and their parents.

## Figures and Tables

**Table 1:** Elements of the implementation support and corresponding ERIC implementation strategies (module 3).

Elements of the Implementation Support	Corresponding ERIC Implementation Strategies
Coaching for the SPC director	Involve executive boards
Coaching for local working groups implementing training content	Identify and prepare champions Organize implementation team meetings Promote adaptability
Team training for new staff members	Conduct ongoing training
Training for administrative staff not covered in module 1	Conduct ongoing training
Online peer exchange across study sites	Create a learning cooperative Capture and share local knowledge Promote network weaving
Refresher training on the *ICF-AddIn*	Conduct ongoing training
Advanced training in motivational interviewing	Conduct ongoing training
Advanced training in ICF-based documen-tation and participation goal setting	Conduct ongoing training

ERIC, Expert Recommendations for Implementing Change.

**Table 2:** Primary and secondary endpoints of the effectiveness evaluation and their operationalisation.

Endpoint	Instrument	Validation Study	Child Survey	Parent Survey
**Cross-sectional component**				
***Primary endpoint***				
Perceived SDM with parents	Parent scale of CollaboRATE^pediatric^	[37]		X
***Secondary endpoints***				
Perceived SDM with patients (if aged 7 years or older)	Paediatric patient scale of CollaboRATE^pediatric^	[37]	X	
Parental satisfaction with care	CHC-SUN short form	[39]		X
Patients’ satisfaction with care (if aged 7 years or older)	YHC-SUN short form	[39]	X	
**Cohort component**				
***Secondary endpoints***				
Participation of patients	CASP	[40]		X
Health-related quality of life of patients	DISABKIDS-10 parent-report scale	[41]		X
	DISABKIDS-10 patient scale	[41]	X	
Utilisation of healthcare services and unmet needs	CHC-SUN long form	[42]		X
Perceived parental stress	PSQ	[43]		X

CASP, Child and Adolescent Scale of Participation; CHC-SUN, Child Health Care–Satisfaction, Utilization and Needs; PSQ, Perceived Stress Questionnaire; SDM, shared decision making; YHC-SUN, Youth Health Care–Satisfaction, Utilization and Needs.

## Supplementary Materials

The following supporting information can be downloaded at: https://www.mdpi.com/article/10.3390/ijerph192416865/s1, File S1: SAS Code for fitting the model for the primary endpoint; File S2: Surveys among SPC directors and their staff; File S3: Guides for semi-structured interviews with SPC directors and their staff; File S4: Completed SPIRIT Checklist; File S5: Consent forms; File S6: Framework for participation-centred care.
