# Supplementary material for: Evaluation of a Complex Intervention to Strengthen Participation-Centred Care for Children with Special Healthcare Needs: Protocol of the Stepped Wedge Cluster Randomised PART-CHILD Trial
Source: Int J Environ Res Public Health. 2022 Dec 15;19(24):16865. doi: 10.3390/ijerph192416865 (PMC9779391; doi:10.3390/ijerph192416865)
Supplement: Supplementary file 1 [file ijerph-19-16865-s001.zip › ijerph-1964850-supplementary.pdf]

Online Supplementary Files

Online Supplementary File S1: SAS Code for fitting the model for the primary endpoint

```
proc glimmix empirical=firores; class SPC PERIOD /ref=first; model
TOPSCORERS/PARENTS = PERIOD INTERVENTION/ oddsratio dist=binomial
ddfm=none covb s; random period / subject=SPC type=cs  residual.
```

Online Supplementary File S2: Surveys among SPC directors and their staff

Fachkräfte-Befragung *(prä-interventionell)*

Bitte geben Sie den ausgefüllten Fragebogen **in den beiliegenden Umschlag, verschließen Sie diesen und legen Sie ihn in die dafür vorgesehene Kiste in Ihrem SPZ-Sekretariat.** Die Fragebögen werden dann gesammelt an das Projektteam weitergeleitet. Ihre Daten werden nur von den Projektmitarbeitern weiterverarbeitet. Die Auswertung erfolgt aggregiert, sodass keine Rückschlüsse auf Ihre Person möglich sind.

I. Patienten- und Elternzentrierung im täglichen Arbeiten

1. Bitte geben Sie an, **inwiefern folgende Aspekte** bei Ihrer Arbeit mit Patienten bzw. Eltern für Sie **wichtig sind**.

☞ *Bitte machen Sie in jeder Zeile ein Kreuz. Wenn Sie als Fachkraft nur mit Kleinkindern bzw. mit schwerbehinderten Patienten arbeiten, kreuzen Sie bitte „nicht zutreffend" an.*

Patienten	**Überhaupt nicht wichtig** 0	1	2	3	4	5	6	7	8	**Sehr wichtig** 9	**Nicht zutreffend**
… bekommen stets klare Informationen.	☐	☐	☐	☐	☐	☐	☐	☐	☐	☐	☐
… bekommen immer Gelegenheit Fragen zu stellen.	☐	☐	☐	☐	☐	☐	☐	☐	☐	☐	☐
… äußern ihre Meinungen.	☐	☐	☐	☐	☐	☐	☐	☐	☐	☐	☐
… werden stets in die Besprechung von Versorgungs- bzw. Therapieoptionen einbezogen.	☐	☐	☐	☐	☐	☐	☐	☐	☐	☐	☐
… werden gleichberechtigt an Entscheidungen über ihre Versorgung bzw. Therapie beteiligt.	☐	☐	☐	☐	☐	☐	☐	☐	☐	☐	☐
… übernehmen die Verantwortung für ihre Gesundheit.	☐	☐	☐	☐	☐	☐	☐	☐	☐	☐	☐

Eltern	**Überhaupt nicht wichtig** 0	1	2	3	4	5	6	7	8	**Sehr wichtig** 9
… bekommen stets klare Informationen.	☐	☐	☐	☐	☐	☐	☐	☐	☐	☐
… bekommen immer Gelegenheit Fragen zu stellen.	☐	☐	☐	☐	☐	☐	☐	☐	☐	☐
… äußern ihre Meinungen.	☐	☐	☐	☐	☐	☐	☐	☐	☐	☐
… werden stets in die Besprechung von Versorgungs- bzw. Therapieoptionen einbezogen.	☐	☐	☐	☐	☐	☐	☐	☐	☐	☐
… werden gleichberechtigt an Entscheidungen über ihre Versorgung bzw. Therapie ihrer Kinder beteiligt.	☐	☐	☐	☐	☐	☐	☐	☐	☐	☐
… übernehmen die Verantwortung für die Gesundheit ihrer Kinder.	☐	☐	☐	☐	☐	☐	☐	☐	☐	☐

2. Bitte geben Sie an, **wie gut Sie in der Regel folgende Aspekte** bei Ihrer Arbeit mit Patienten bzw. Eltern **umsetzen können**.

☞ *Bitte machen Sie in jeder Zeile ein Kreuz. Wenn Sie als Fachkraft nur mit Kleinkindern bzw. mit schwerbehinderten Patienten arbeiten, kreuzen Sie bitte „nicht zutreffend" an.*

Patienten	**Überhaupt nicht gut** 0	1	2	3	4	5	6	7	8	**Sehr gut** 9	**Nicht zutreffend**
… bekommen stets klare Informationen.	☐	☐	☐	☐	☐	☐	☐	☐	☐	☐	☐
… bekommen immer Gelegenheit Fragen zu stellen.	☐	☐	☐	☐	☐	☐	☐	☐	☐	☐	☐
… äußern ihre Meinungen.	☐	☐	☐	☐	☐	☐	☐	☐	☐	☐	☐
… werden stets in die Besprechung von Versorgungs- bzw. Therapieoptionen einbezogen.	☐	☐	☐	☐	☐	☐	☐	☐	☐	☐	☐
… werden gleichberechtigt an Entscheidungen über ihre Versorgung bzw. Therapie beteiligt.	☐	☐	☐	☐	☐	☐	☐	☐	☐	☐	☐
… übernehmen die Verantwortung für ihre Gesundheit.	☐	☐	☐	☐	☐	☐	☐	☐	☐	☐	☐

Eltern	**Überhaupt nicht gut** 0	1	2	3	4	5	6	7	8	**Sehr gut** 9
… bekommen stets klare Informationen.	☐	☐	☐	☐	☐	☐	☐	☐	☐	☐
… bekommen immer Gelegenheit Fragen zu stellen.	☐	☐	☐	☐	☐	☐	☐	☐	☐	☐
… äußern ihre Meinungen.	☐	☐	☐	☐	☐	☐	☐	☐	☐	☐
… werden stets in die Besprechung von Versorgungs- bzw. Therapieoptionen einbezogen.	☐	☐	☐	☐	☐	☐	☐	☐	☐	☐
… werden gleichberechtigt an Entscheidungen über ihre Versorgung bzw. Therapie ihrer Kinder beteiligt.	☐	☐	☐	☐	☐	☐	☐	☐	☐	☐
… übernehmen die Verantwortung für die Gesundheit ihrer Kinder.	☐	☐	☐	☐	☐	☐	☐	☐	☐	☐

3. Welche Barrieren **erschweren** Ihrer Meinung nach die **Patienten- und Elternzentrierung** in Ihrem beruflichen Alltag?

☞ *Bitte nennen Sie drei aus Ihrer Sicht gravierende Barrieren. Stichpunkte reichen aus.*

1.

2.

3.

II. Merkmale des Arbeitsumfelds SPZ

1. Eines der Ziele des Projekts PART-CHILD ist die Umsetzung **einer teilhabeorientierten Patientenversorgung durch die Implementierung der ICF-CY in Ihrem SPZ.** Die teilhabeorientierte Patientenversorgung wird in den folgenden Aussagen kurz als **„Veränderung"** bezeichnet.

Bitte schätzen Sie, inwiefern diese Aussagen für Ihr SPZ zutreffen werden.

☞ *Bitte machen Sie in jeder Zeile ein Kreuz.*

	**Trifft überhaupt nicht zu**	**Trifft eher nicht zu**	**Trifft teilweise zu**	**Trifft eher zu**	**Trifft voll und ganz zu**
Die Leitung des SPZ kann die Mitarbeiter zur Umsetzung der Veränderung überdurchschnittlich gut motivieren.	☐	☐	☐	☐	☐
Der Leitung des SPZ ist es ein Anliegen, dass die Veränderung umgesetzt wird.	☐	☐	☐	☐	☐
Die Leitung des SPZ wird die Umsetzung der Veränderung systematisch unterstützen.	☐	☐	☐	☐	☐
Alle Mitarbeiter werden sich bei der Umsetzung der Veränderung engagieren.	☐	☐	☐	☐	☐
Alle Mitarbeiter sind zuversichtlich, dass sie mit einer stärkeren Ausrichtung der Patientenversorgung an Teilhabezielen Schritt halten können.	☐	☐	☐	☐	☐
Alle Mitarbeiter werden alles tun, was nötig ist, damit die Umsetzung der Veränderung gelingen kann.	☐	☐	☐	☐	☐
Alle Mitarbeiter sind zuversichtlich, dass das SPZ insgesamt (inkl. Geschäftsführung, IT) sie bei der Umsetzung der Veränderung unterstützen wird.	☐	☐	☐	☐	☐
Alle Mitarbeiter wünschen sich, dass die Veränderung umgesetzt wird.	☐	☐	☐	☐	☐
Alle Mitarbeiter sind zuversichtlich, dass die bei der Umsetzung der Veränderung entstehende Dynamik anhalten wird.	☐	☐	☐	☐	☐
Alle Mitarbeiter sind zuversichtlich, die Herausforderungen bewältigen zu können, die während der Umsetzung der Veränderung aufkommen könnten.	☐	☐	☐	☐	☐
Alle Mitarbeiter sind entschlossen, die Veränderung umzusetzen.	☐	☐	☐	☐	☐
Alle Mitarbeiter sind zuversichtlich, die im Zuge der Veränderung entstehenden Aufgaben koordinieren zu können.	☐	☐	☐	☐	☐
Alle Mitarbeiter sind motiviert, die Veränderung umzusetzen.	☐	☐	☐	☐	☐
Alle Mitarbeiter sind zuversichtlich, die ***einrichtungs-politischen*** Herausforderungen innerhalb der Organisation bewältigen zu können, die die Veränderung mit sich bringt (z.B. abteilungsübergreifende Zusammenarbeit innerhalb der Klinik).	☐	☐	☐	☐	☐
Alle Mitarbeiter sind zuversichtlich, die ***einrichtungs-übergreifenden*** Herausforderungen bewältigen zu können, die die Veränderung mit sich bringt (z.B. Zusammenarbeit mit Kindergärten, Schulen).	☐	☐	☐	☐	☐

2. Folgende Fragen beziehen sich auf die Kultur in Ihrer Einrichtung. Die Unterpunkte enthalten jeweils vier Beschreibungen. Bitte verteilen Sie 100 Punkte auf vier Beschreibungen, je nachdem, wie gut jede Beschreibung auf Ihre Einrichtung zutrifft. Jede Beschreibung ist anders und keine ist besser oder schlechter als die anderen.

Zum Beispiel: In Frage 2.1. trifft Beschreibung A sehr gut auf Ihre Einrichtung zu, B trifft etwas zu und C und D treffen überhaupt nicht zu. Sie können der Beschreibung A 70 Punkte und der Beschreibung B 30 Punkte zuordnen.

A. ___70
B. ___30
C. ____0
D. ____0

2.1. Meine Einrichtung …

☞ *Bitte verteilen Sie insgesamt 100 Punkte.*

A.	… hat einen sehr *persönlichen Charakter*. Sie ist wie eine große Familie. Die Mitarbeiter scheinen viel mit einander zu teilen.	___________
B.	… ist sehr *dynamisch und unternehmerisch*. Die Mitarbeiter sind bereit, etwas zu wagen.	___________
C.	… orientiert sich an *Regeln und ist gut strukturiert*. In der Regel orientieren sich die Handlungen der Mitarbeiter an festgelegten Prozessen und Verfahren.	___________
D.	… ist sehr *ergebnisorientiert*. Ein Hauptanliegen besteht darin, die Arbeit zu erledigen. Die Mitarbeiter zeigen kaum persönliches Engagement bei der Umsetzung von Aufgaben.	___________
		Σ 100 Punkte

2.2. Die Mitarbeiter sehen in Ihrer SPZ-Leitung …

☞ *Bitte verteilen Sie insgesamt 100 Punkte.*

A.	… einen Mentor, der seine Mitarbeiter fürsorglich unterstützt.	___________
B.	… jemanden, der unternehmerisch, innovativ oder risikofreudig ist.	___________
C.	… einen Koordinator, Organisator oder Administrator.	___________
D.	… einen Produzenten, Techniker oder Antreiber.	___________
		Σ 100 Punkte

2.3. Meine Einrichtung wird zusammengehalten durch...

☞ *Bitte verteilen Sie insgesamt 100 Punkte.*

A.	… Loyalität und Tradition. Der persönliche Einsatz ist groß.	___________
B.	… Freude an Innovation und Entwicklung. Nahe am Puls der Zeit zu sein ist sehr wichtig.	___________
C.	… formale Regeln und Richtlinien. Hier ist es wichtig reibungslose Abläufe aufrechtzuerhalten.	___________
D.	… eine ergebnisorientierte Haltung. Bei den Mitarbeitern stehen Aufgaben und Zielerreichung im Vordergrund.	___________
		Σ 100 Punkte

2.4. Meine Einrichtung legt Wert auf…

☞ *Bitte verteilen Sie insgesamt 100 Punkte.*

A.	… menschliche Ressourcen. Mitarbeiterzusammenhalt und Leitkultur sind wichtig.	___________
B.	… Wachstum und die Erschließung neuer Arbeits- und Therapiefelder. Die Bereitschaft, sich neuen Herausforderungen zu stellen, ist wichtig.	___________
C.	… Beständigkeit und Stabilität. Effiziente und reibungslose Abläufe sind wichtig.	___________
D.	… Wettbewerbsfähigkeit und Leistung. Messbare Ziele sind wichtig.	___________
		Σ 100 Punkte

III. Teilhabeorientierte Versorgung im Arbeitsumfeld SPZ

1. Die **teilhabeorientierte Patientenversorgung durch die Implementierung der ICF-CY** wird auch in den folgenden Aussagen kurz als **„Veränderung“** bezeichnet.
Bitte schätzen Sie, inwiefern diese Aussagen auf Ihr SPZ zutreffen.
☞ *Bitte machen Sie in jeder Zeile ein Kreuz.*

	Trifft überhaupt nicht zu	Trifft eher nicht zu	Trifft teilweise zu	Trifft eher zu	Trifft voll und ganz zu
Die Mitarbeiter haben das Gefühl, dass die Veränderung mit den Werten zusammenpasst, die im SPZ gelebt werden.	☐	☐	☐	☐	☐
Die Mitarbeiter brauchen diese Veränderung im SPZ.	☐	☐	☐	☐	☐
Die Mitarbeiter glauben, dass diese Veränderung im SPZ unerlässlich ist.	☐	☐	☐	☐	☐
Die Mitarbeiter glauben, dass die Umsetzung dieser Veränderung im SPZ gelingen wird.	☐	☐	☐	☐	☐
Die Mitarbeiter betrachten diese Veränderung als zeitgemäß.	☐	☐	☐	☐	☐
Die Mitarbeiter glauben, dass die Umsetzung dieser Veränderung in einem adäquaten Kosten-Nutzen-Verhältnis steht.	☐	☐	☐	☐	☐
Die Mitarbeiter glauben, dass sich die Gesamtsituation im SPZ durch diese Veränderung verbessern wird.	☐	☐	☐	☐	☐
Die Mitarbeiter haben das Gefühl, dass die Umsetzung dieser Veränderung eine gute Idee ist.	☐	☐	☐	☐	☐
Die Mitarbeiter wertschätzen diese Veränderung.	☐	☐	☐	☐	☐

IV. Allgemeine Angaben zur Patientenversorgung

1. Wie viele Patientenkontakte haben Sie derzeit durchschnittlich pro Woche?

Präsenztermine: |__|__|__|, davon dauern:
|__|__|__| über 2 Stunden
|__|__|__| 1 bis 2 Stunden
|__|__|__| unter 1 Stunde

Telefonische/ E-Mail-Kontakte: |__|__|__|

2. Wie hoch ist Ihre derzeitige durchschnittliche Gesamtarbeitszeit pro Woche (inklusive evtl. anfallender Überstunden)? |__|__| Stunden

3. Wenn Sie an Ihre durchschnittliche wöchentliche Arbeitszeit im SPZ denken (inklusive evtl. anfallender Überstunden): Wie viele Stunden wenden Sie gegenwärtig für folgende Tätigkeiten auf:

Die konkrete Arbeit mit Patienten und Eltern?	______ Stunden
Die Therapieplanung (inkl. Vorbereitung und Terminvereinbarung)?	______ Stunden
Die Dokumentation (z.B. Diagnosen, Befunde, Arztberichte, Bescheinigungen)?	______ Stunden
Die fallbezogene interdisziplinäre Zusammenarbeit im SPZ (z.B. Fallkonferenzen, runde Tische)?	______ Stunden
Die einrichtungsübergreifende Zusammenarbeit (z.B. mit Jugend-/ Sozialamt)?	______ Stunden
Die Weiterentwicklung der Versorgung am SPZ (z.B. Arbeitsgruppe zur Umsetzung neuer Standards)?	______ Stunden

sonstige Tätigkeiten, z.B.:	____________________	______ Stunden
	____________________	______ Stunden
	____________________	______ Stunden

4. Wie effizient schätzen Sie derzeit …

☞ *Bitte machen Sie in jeder Zeile ein Kreuz auf der Skala.*

	Überhaupt nicht effizient 0	1	2	3	4	5	6	7	8	Sehr effizient 9	Kann nicht beurteilen
… die konkrete Arbeit mit Patienten und Eltern in Ihrem SPZ ein?	☐	☐	☐	☐	☐	☐	☐	☐	☐	☐	☐
… die Therapieplanung (inkl. Vorbereitung und Terminvereinbarung) ein?	☐	☐	☐	☐	☐	☐	☐	☐	☐	☐	☐
… die Dokumentation in Ihrem SPZ ein?	☐	☐	☐	☐	☐	☐	☐	☐	☐	☐	☐
… die ***fallbezogene*** interdisziplinäre Zusammenarbeit in Ihrem SPZ ein?	☐	☐	☐	☐	☐	☐	☐	☐	☐	☐	☐
… die ***fallunabhängige*** interdisziplinäre Zusammenarbeit in Ihrem SPZ (z.B. Erweiterung von Therapiestandards) ein?	☐	☐	☐	☐	☐	☐	☐	☐	☐	☐	☐
… die ***einrichtungsübergreifende*** Zusammenarbeit (z.B.Kinderärzte, Jugend-/Sozialamt, Frühförder-stellen) ein?	☐	☐	☐	☐	☐	☐	☐	☐	☐	☐	☐

5. Bitte denken Sie an das letzte Quartal: Wie hoch war der Anteil Ihrer Patienten und Eltern, die sich ziemlich genau an getroffene Vereinbarungen gehalten haben bezüglich:

☞ *Bitte machen Sie in jeder Zeile ein Kreuz auf der Skala.*
Wenn ein Punkt für Ihre Berufsgruppe nicht beurteilbar ist, kreuzen Sie „Kann nicht beurteilen" an.

	0% – 10% – 20% – 30% – 40% – 50% – 60% – 70% – 80% – 90% – 100%	Kann nicht beurteilen
Einnahme verordneter Medikamente?	|—————————————————————|	☐
Inanspruchnahme von sonstigen nicht-ärztlichen Leistungen?		
Ergotherapie	|—————————————————————|	☐
Logopädie	|—————————————————————|	☐
Physiotherapie	|—————————————————————|	☐
Sonstige Therapien: ____________________	|—————————————————————|	☐
Ausstattung mit Hilfsmitteln (z.B. Gehhilfen, Schreibhilfen, Rollstuhl)?	|—————————————————————|	☐
Inanspruchnahme von Schulungen bzw. Beratungen (z.B. Elterntraining, Beratung zur Erziehung, sozialen Hilfen)?	0% – 10% – 20% – 30% – 40% – 50% – 60% – 70% – 80% – 90% – 100% |—————————————————————|	☐

V. Allgemeine Zufriedenheit mit der beruflichen Tätigkeit

☞ *Bitte machen Sie in jeder Zeile ein Kreuz.*

	Stimme überhaupt nicht zu 0	1	2	3	4	5	6	7	8	Stimme voll und ganz zu 9
Ich persönlich empfinde meine derzeitige Arbeit im SPZ als lohnend.	☐	☐	☐	☐	☐	☐	☐	☐	☐	☐
Insgesamt gefällt mir meine Arbeit hier im SPZ.	☐	☐	☐	☐	☐	☐	☐	☐	☐	☐
Insgesamt bin ich mit meiner derzeitigen Arbeit zufrieden.	☐	☐	☐	☐	☐	☐	☐	☐	☐	☐
Meine derzeitige Arbeitssituation bietet für mich großen Anlass zur Frustration.	☐	☐	☐	☐	☐	☐	☐	☐	☐	☐
Meine Arbeit im SPZ entspricht nicht meinen Erwartungen.	☐	☐	☐	☐	☐	☐	☐	☐	☐	☐
Ich empfinde meine derzeitige Arbeit im SPZ als sinnstiftend.	☐	☐	☐	☐	☐	☐	☐	☐	☐	☐
Meine derzeitige Arbeitssituation ist durch Zeitdruck geprägt.	☐	☐	☐	☐	☐	☐	☐	☐	☐	☐
Meine derzeitige Arbeitssituation ist durch Kostendruck geprägt.	☐	☐	☐	☐	☐	☐	☐	☐	☐	☐
In meiner Arbeit kann ich viele Entscheidungen selbst treffen.	☐	☐	☐	☐	☐	☐	☐	☐	☐	☐
Ich habe sehr wenig Entscheidungsspielraum darüber, wie ich meine Arbeit erledige.	☐	☐	☐	☐	☐	☐	☐	☐	☐	☐

VI. Angaben zu Ihrer Person

1. Ihr Geschlecht

☐ Männlich ☐ Weiblich

2. Bitte geben Sie an, in welchem Fachbereich Sie tägig sind:

☐ Kinderheilkunde
☐ Kinder- und Jugendpsychiatrie
☐ Pädagogik
☐ Heilpädagogik
☐ Psychologie
☐ Psychotherapie
☐ Ergotherapie
☐ Physiotherapie
☐ Logopädie
☐ Sonstiges: ________________________________

3. Ihre Erfahrung in Ihrem derzeitigen Fachbereich: |__|__| Jahre

4. Bitte geben Sie Ihren höchsten beruflichen Ausbildungsabschluss an:

☐ Promotion
☐ Master/ Magister/ Staatsexamen
☐ Diplom
☐ Bachelor, Diplom FH
☐ Beruflich-schulische Ausbildung (Berufsfachschule)
☐ Beruflich-betriebliche Berufsausbildung (Lehre)
☐ Sonstiges: ________________________________

5. Haben Sie die ICF-CY in der Vergangenheit bereits angewendet?

- ☐ Nein
- ☐ Ich habe von der ICF-CY schon einmal gehört
- ☐ Ich habe die ICF-CY schon einmal angewendet
- ☐ Ich wende die ICF-CY gelegentlich an
- ☐ Ich wende die ICF-CY regelmäßig an
- ☐ Ich wende die ICF-CY immer an

☞ *Bitte beschreiben Sie hier kurz Ihre bisherigen Erfahrungen mit der ICF-CY*

6. Sind Sie außerhalb der Patientenversorgung mit weiteren (formellen oder informellen) Aufgaben im SPZ betraut?
(z.B. Sprecherin einer Arbeitsgruppe, Organisation bzw. Aufbau eines neuen Bereichs, Supervision von EEG)

- ☐ Ja, und zwar: ____
- ☐ Nein

Zum Schluss geben Sie bitte Ihren Code an, den Sie gewählt haben

Zur Erinnerung:

In die ersten zwei Kästchen notieren Sie die **zwei letzten Buchstaben des ersten Vornamens Ihrer Mutter**.
In die nächsten zwei Kästchen notieren Sie die **zwei letzten Buchstaben des Nachnamens Ihrer Mutter**.
In die folgenden zwei Kästchen notieren **Sie den Geburtstag Ihrer Mutter**.
In die letzten zwei Kästchen notieren Sie **den Geburtsmonat Ihrer Mutter**.

Beispiel: Ihre Mutter heißt Pet**ra** Johanna Musterfr**au**. Ihre Mutter hat am **09.04.** Geburtstag.

Der Code lautet daher: R A A U 0 9 0 4

Vielen Dank für Ihre Teilnahme!

Bitte geben Sie den ausgefüllten Fragebogen **in den beiliegenden Umschlag, verschließen Sie diesen und legen Sie ihn in die dafür vorgesehene Kiste in Ihrem SPZ-Sekretariat.** Die Fragebögen werden dann gesammelt an das Projektteam weitergeleitet. Ihre Daten werden nur von den Projektmitarbeitern weiterverarbeitet. Die Auswertung erfolgt aggregiert, sodass keine Rückschlüsse auf Ihre Person möglich sind.

Befragung der SPZ-Leitung *(prä-interventionell)*

Bitte geben Sie den ausgefüllten Fragebogen **in den beiliegenden Umschlag, verschließen Sie diesen** und schicken Sie ihn zusammen mit den Fragebögen Ihrer Mitarbeiter an das Projektteam. Ihre Daten werden nur von den Projektmitarbeitern weiterverarbeitet. Die Auswertung erfolgt aggregiert, sodass keine Rückschlüsse auf Ihre Person möglich sind.

I. Patienten- und Elternzentrierung im täglichen Arbeiten

1. Bitte geben Sie an, **inwiefern folgende Aspekte** in Ihrer Arbeit mit Patienten bzw. Eltern für Sie **wichtig sind**.

☞ *Bitte machen Sie in jeder Zeile ein Kreuz. Wenn Sie als Fachkraft nur mit Kleinkindern bzw. mit schwerbehinderten Patienten arbeiten, kreuzen Sie bitte „nicht zutreffend" an.*

	**Überhaupt nicht wichtig**									**Sehr wichtig**	**Nicht zutreffend**
Patienten	0	1	2	3	4	5	6	7	8	9	
… bekommen stets klare Informationen.	☐	☐	☐	☐	☐	☐	☐	☐	☐	☐	☐
… bekommen immer Gelegenheit, Fragen zu stellen.	☐	☐	☐	☐	☐	☐	☐	☐	☐	☐	☐
… äußern ihre Meinungen.	☐	☐	☐	☐	☐	☐	☐	☐	☐	☐	☐
… werden stets in die Besprechung von Versorgungs- bzw. Therapieoptionen einbezogen.	☐	☐	☐	☐	☐	☐	☐	☐	☐	☐	☐
… werden gleichberechtigt an Entscheidungen über ihre Versorgung bzw. Therapie beteiligt.	☐	☐	☐	☐	☐	☐	☐	☐	☐	☐	☐
… übernehmen die Verantwortung für ihre Gesundheit.	☐	☐	☐	☐	☐	☐	☐	☐	☐	☐	☐

	**Überhaupt nicht wichtig**									**Sehr wichtig**
Eltern	0	1	2	3	4	5	6	7	8	9
… bekommen stets klare Informationen.	☐	☐	☐	☐	☐	☐	☐	☐	☐	☐
… bekommen immer Gelegenheit, Fragen zu stellen.	☐	☐	☐	☐	☐	☐	☐	☐	☐	☐
… äußern ihre Meinungen.	☐	☐	☐	☐	☐	☐	☐	☐	☐	☐
… werden stets in die Besprechung von Versorgungs- bzw. Therapieoptionen einbezogen.	☐	☐	☐	☐	☐	☐	☐	☐	☐	☐
… werden gleichberechtigt an Entscheidungen über ihre Versorgung bzw. Therapie ihrer Kinder beteiligt.	☐	☐	☐	☐	☐	☐	☐	☐	☐	☐
… übernehmen die Verantwortung für die Gesundheit ihrer Kinder.	☐	☐	☐	☐	☐	☐	☐	☐	☐	☐

2. Bitte geben Sie an, **wie gut Sie in der Regel folgende Aspekte** in Ihrer Arbeit mit Patienten bzw. Eltern **umsetzen können**.

☞ *Bitte machen Sie in jeder Zeile ein Kreuz. Wenn Sie als Fachkraft nur mit Kleinkindern bzw. mit schwerbehinderten Patienten arbeiten, kreuzen Sie bitte „nicht zutreffend" an.*

Patienten	**Überhaupt nicht gut** 0	1	2	3	4	5	6	7	8	**Sehr gut** 9	**Nicht zutreffend**
… bekommen stets klare Informationen.	☐	☐	☐	☐	☐	☐	☐	☐	☐	☐	☐
… bekommen immer Gelegenheit Fragen zu stellen.	☐	☐	☐	☐	☐	☐	☐	☐	☐	☐	☐
… äußern ihre Meinungen.	☐	☐	☐	☐	☐	☐	☐	☐	☐	☐	☐
… werden stets in die Besprechung von Versorgungs- bzw. Therapieoptionen einbezogen.	☐	☐	☐	☐	☐	☐	☐	☐	☐	☐	☐
… werden gleichberechtigt an Entscheidungen über ihre Versorgung bzw. Therapie beteiligt.	☐	☐	☐	☐	☐	☐	☐	☐	☐	☐	☐
… übernehmen die Verantwortung für ihre Gesundheit.	☐	☐	☐	☐	☐	☐	☐	☐	☐	☐	☐

Eltern	**Überhaupt nicht gut** 0	1	2	3	4	5	6	7	8	**Sehr gut** 9
… bekommen stets klare Informationen.	☐	☐	☐	☐	☐	☐	☐	☐	☐	☐
… bekommen immer Gelegenheit Fragen zu stellen.	☐	☐	☐	☐	☐	☐	☐	☐	☐	☐
… äußern ihre Meinungen.	☐	☐	☐	☐	☐	☐	☐	☐	☐	☐
… werden stets in die Besprechung von Versorgungs- bzw. Therapieoptionen einbezogen.	☐	☐	☐	☐	☐	☐	☐	☐	☐	☐
… werden gleichberechtigt an Entscheidungen über die Versorgung bzw. Therapie ihrer Kinder beteiligt.	☐	☐	☐	☐	☐	☐	☐	☐	☐	☐
… übernehmen die Verantwortung für die Gesundheit ihrer Kinder.	☐	☐	☐	☐	☐	☐	☐	☐	☐	☐

3. Welche Barrieren **erschweren** Ihrer Meinung nach die **Patienten- und Elternzentrierung** in Ihrem beruflichen Alltag?

☞ *Bitte nennen Sie drei aus Ihrer Sicht gravierende Barrieren. Stichpunkte reichen aus.*

1.

2.

3.

II. Merkmale des Arbeitsumfelds SPZ

1. Eines der Ziele des Projekts PART-CHILD ist die Umsetzung **einer teilhabeorientierten Patientenversorgung durch die Implementierung der ICF-CY in Ihrem SPZ.** Die teilhabeorientierte Patientenversorgung wird in den folgenden Aussagen kurz als **„Veränderung“** bezeichnet.

Bitte schätzen Sie, inwiefern diese Aussagen auf Sie als SPZ-Leitung bzw. auf alle SPZ-Mitarbeiter zutreffen werden.
☞ *Bitte machen Sie in jeder Zeile ein Kreuz.*

	Trifft überhaupt nicht zu	Trifft eher nicht zu	Trifft teilweise zu	Trifft eher zu	Trifft voll und ganz zu
Sie als SPZ-Leitung können die Mitarbeiter zur Umsetzung der Veränderung motivieren.	☐	☐	☐	☐	☐
Es ist Ihnen als SPZ-Leitung eines der wichtigsten Anliegen, dass die Veränderung umgesetzt wird.	☐	☐	☐	☐	☐
Sie als SPZ-Leitung haben alle notwendigen Ressourcen (z.B. Erfahrung, organisationale Gegebenheiten), damit die Veränderung in Ihrem SPZ umgesetzt werden kann.	☐	☐	☐	☐	☐
Alle Mitarbeiter werden sich bei der Umsetzung der Veränderung engagieren.	☐	☐	☐	☐	☐
Alle Mitarbeiter sind zuversichtlich, dass sie mit einer stärkeren Ausrichtung der Patientenversorgung an Teilhabezielen Schritt halten können.	☐	☐	☐	☐	☐
Alle Mitarbeiter werden alles tun, was nötig ist, damit die Umsetzung der Veränderung gelingen kann.	☐	☐	☐	☐	☐
Alle Mitarbeiter sind zuversichtlich, dass ihre Organisation insgesamt (inkl. Geschäftsführung, IT) sie bei der Umsetzung der Veränderung unterstützen wird.	☐	☐	☐	☐	☐
Alle Mitarbeiter sind zuversichtlich, dass alle notwendigen Ressourcen zur Verfügung stehen (z.B. Know-How, Erfahrung, Zeit), damit die Veränderung im SPZ umgesetzt werden kann.	☐	☐	☐	☐	☐
Alle Mitarbeiter wünschen sich, dass die Veränderung umgesetzt wird.	☐	☐	☐	☐	☐
Alle Mitarbeiter sind zuversichtlich, dass die bei der Umsetzung der Veränderung entstehende Dynamik anhalten wird.	☐	☐	☐	☐	☐
Alle Mitarbeiter sind zuversichtlich, die Herausforderungen bewältigen zu können, die während der Umsetzung der Veränderung aufkommen könnten.	☐	☐	☐	☐	☐
Alle Mitarbeiter sind entschlossen, die Veränderung umzusetzen.	☐	☐	☐	☐	☐
Alle Mitarbeiter sind zuversichtlich, die im Zuge der Veränderung entstehenden Aufgaben koordinieren zu können.	☐	☐	☐	☐	☐
Alle Mitarbeiter sind motiviert, die Veränderung umzusetzen.	☐	☐	☐	☐	☐

	Trifft überhaupt nicht zu	Trifft eher nicht zu	Trifft teilweise zu	Trifft eher zu	Trifft voll und ganz zu

Alle Mitarbeiter sind zuversichtlich, die ***einrichtungs-politischen*** Herausforderungen innerhalb der Organisation bewältigen zu können, die die Veränderung mit sich bringt (z.B. abteilungsübergreifende Zusammenarbeit innerhalb der Klinik).	☐	☐	☐	☐	☐
Alle Mitarbeiter sind zuversichtlich, die ***einrichtungs-übergreifenden*** Herausforderungen bewältigen zu können, die die Veränderung mit sich bringt (z.B. Zusammenarbeit mit Kindergärten, Schulen).	☐	☐	☐	☐	☐
Der Geschäftsführung ist es ein Anliegen, dass die Veränderung umgesetzt wird.	☐	☐	☐	☐	☐
Die übergeordneten Ebenen (z.B. Geschäftsführung, Verwaltung) unterstützen die Umsetzung der Veränderung.	☐	☐	☐	☐	☐
Der IT-Bereich unterstützt die Umsetzung der Veränderung.	☐	☐	☐	☐	☐

2. Folgende Fragen beziehen sich auf die Kultur in Ihrer Einrichtung. Die Unterpunkte enthalten jeweils vier Beschreibungen. Bitte verteilen Sie 100 Punkte auf vier Beschreibungen, je nachdem, wie gut jede Beschreibung auf Ihre Einrichtung zutrifft. Jede Beschreibung ist anders und keine ist besser oder schlechter als die anderen.

Zum Beispiel: In Frage 2.1. trifft Beschreibung A sehr gut auf Ihre Einrichtung zu, B trifft etwas zu und C und D treffen überhaupt nicht zu. Sie können der Beschreibung A 70 Punkte und der Beschreibung B 30 Punkte zuordnen.

A. ___70
B. ___30
C. ____0
D. ____0

2.1. Meine Einrichtung …

☞ *Bitte verteilen Sie insgesamt 100 Punkte.*

A.	… hat einen sehr *persönlichen Charakter*. Sie ist wie eine große Familie. Die Mitarbeiter scheinen viel mit einander zu teilen.	__________
B.	… ist sehr *dynamisch und unternehmerisch*. Die Mitarbeiter sind bereit, etwas zu wagen.	__________
C.	… orientiert sich an *Regeln und ist gut strukturiert*. In der Regel orientieren sich die Handlungen der Mitarbeiter an festgelegten Prozessen und Verfahren.	__________
D.	… ist sehr *ergebnisorientiert*. Ein Hauptanliegen besteht darin, die Arbeit zu erledigen. Die Mitarbeiter zeigen kaum persönliches Engagement bei der Umsetzung von Aufgaben.	__________
		Σ 100 Punkte

2.2. Die Mitarbeiter sehen in Ihrer SPZ-Leitung …

☞ *Bitte verteilen Sie insgesamt 100 Punkte.*

A.	… einen Mentor, der seine Mitarbeiter fürsorglich unterstützt.	__________
B.	… jemanden, der unternehmerisch, innovativ oder risikofreudig ist.	__________
C.	… einen Koordinator, Organisator oder Administrator.	__________
D.	… einen Produzenten, Techniker oder Antreiber.	__________
		Σ 100 Punkte

2.3. Meine Einrichtung wird zusammengehalten durch...

☞ *Bitte verteilen Sie insgesamt 100 Punkte.*

A.	… Loyalität und Tradition. Der persönliche Einsatz ist groß.	__________

B.	… Freude an Innovation und Entwicklung. Am Puls der Zeit zu sein ist sehr wichtig.	___________
C.	… formale Regeln und Richtlinien. Hier ist es wichtig reibungslose Abläufe aufrechtzuerhalten.	___________
D.	… eine ergebnisorientierte Haltung. Bei den Mitarbeitern stehen Aufgaben und Zielerreichung im Vordergrund.	___________
		Σ 100 Punkte

2.4. Meine Einrichtung legt Wert auf…

☞ *Bitte verteilen Sie insgesamt 100 Punkte.*

A.	… menschliche Ressourcen. Mitarbeiterzusammenhalt und Leitkultur sind wichtig.	___________
B.	… Innovation und die Erschließung neuer Arbeits- und Therapiefelder. Die Bereitschaft, sich neuen Herausforderungen zu stellen, ist wichtig.	___________
C.	… Beständigkeit und Stabilität. Effiziente und reibungslose Abläufe sind wichtig.	___________
D.	… Wettbewerbsfähigkeit und Leistung. Messbare Ziele sind wichtig.	___________
		Σ 100 Punkte

3. Wie viele Fachkräfte, die diagnostisch, therapeutisch oder beratend tätig sind, sind derzeit in Ihrem SPZ angestellt?

☞ *Bitte geben Sie die Anzahl a) in Köpfen und b) in Vollzeitäquivalenten (VÄ) an, z.B. 3 Köpfe und 2,5 VÄ für 2 Vollzeitkräfte und eine 50%-Kraft*

	Köpfe	VÄ		Köpfe	VÄ
Fachärzte für Kinder- und Jugendmedizin	________	________	Logopäden	________	________
Fachärzte für Kinder- und Jugendpsychiatrie	________	________	Heilpädagogen	________	________
Assistenzärzte in Weiterbildung	________	________	Pädagogen	________	________
Psychologen	________	________	Psychotherapeuten	________	________
Physiotherapeuten	________	________	weitere Therapeuten insgesamt (z.B. Motologie, Musiktherapie)	________	________
Ergotherapeuten	________	________			

III. Teilhabeorientierte Versorgung im Arbeitsumfeld SPZ

1. Die **teilhabeorientierte Patientenversorgung durch die Implementierung der ICF-CY** wird auch in den folgenden Aussagen kurz als **„Veränderung"** bezeichnet.
Bitte schätzen Sie, inwiefern diese Aussagen auf Ihr SPZ zutreffen.

☞ *Bitte machen Sie in jeder Zeile ein Kreuz.*

	Trifft überhaupt nicht zu	Trifft eher nicht zu	Trifft teilweise zu	Trifft eher zu	Trifft voll und ganz zu
Die Mitarbeiter haben das Gefühl, dass die Veränderung mit den Werten zusammenpasst, die an unserem SPZ gelebt werden.	☐	☐	☐	☐	☐
Die Mitarbeiter brauchen diese Veränderung im SPZ.	☐	☐	☐	☐	☐

	Trifft überhaupt nicht zu	Trifft eher nicht zu	Trifft teilweise zu	Trifft eher zu	Trifft voll und ganz zu
Die Mitarbeiter glauben, dass diese Veränderung im SPZ unerlässlich ist.	☐	☐	☐	☐	☐

Die Mitarbeiter glauben, dass die Umsetzung dieser Veränderung im SPZ gelingen wird.	☐	☐	☐	☐	☐
Die Mitarbeiter betrachten diese Veränderung als zeitgemäß.	☐	☐	☐	☐	☐
Die Mitarbeiter glauben, dass die Umsetzung dieser Veränderung in einem adäquaten Kosten-Nutzen-Verhältnis steht.	☐	☐	☐	☐	☐
Die Mitarbeiter glauben, dass sich die Gesamtsituation im SPZ durch diese Veränderung verbessern wird.	☐	☐	☐	☐	☐
Die Mitarbeiter haben das Gefühl, dass die Umsetzung dieser Veränderung eine gute Idee ist.	☐	☐	☐	☐	☐
Die Mitarbeiter wertschätzen diese Veränderung.	☐	☐	☐	☐	☐

IV. Allgemeine Zufriedenheit mit der beruflichen Tätigkeit

☞ *Bitte machen Sie in jeder Zeile ein Kreuz.*

	**Stimme überhaupt nicht zu** 0	1	2	3	4	5	6	7	8	**Stimme voll und ganz zu** 9
Ich persönlich empfinde meine derzeitige Arbeit im SPZ als lohnend.	☐	☐	☐	☐	☐	☐	☐	☐	☐	☐
Insgesamt gefällt mir meine Arbeit hier im SPZ.	☐	☐	☐	☐	☐	☐	☐	☐	☐	☐
Insgesamt bin ich mit meiner derzeitigen Arbeit zufrieden.	☐	☐	☐	☐	☐	☐	☐	☐	☐	☐
Meine derzeitige Arbeitssituation bietet für mich großen Anlass zur Frustration.	☐	☐	☐	☐	☐	☐	☐	☐	☐	☐
Meine Arbeit im SPZ entspricht nicht meinen Erwartungen.	☐	☐	☐	☐	☐	☐	☐	☐	☐	☐
Ich empfinde meine derzeitige Arbeit im SPZ als sinnstiftend.	☐	☐	☐	☐	☐	☐	☐	☐	☐	☐
Meine derzeitige Arbeitssituation ist durch Zeitdruck geprägt.	☐	☐	☐	☐	☐	☐	☐	☐	☐	☐
Meine derzeitige Arbeitssituation ist durch Kostendruck geprägt.	☐	☐	☐	☐	☐	☐	☐	☐	☐	☐
In meiner Arbeit kann ich viele Entscheidungen selbst treffen.	☐	☐	☐	☐	☐	☐	☐	☐	☐	☐
Ich habe sehr wenig Entscheidungsspielraum darüber, wie ich meine Arbeit erledige.	☐	☐	☐	☐	☐	☐	☐	☐	☐	☐

V. Ihre Erfahrungen mit Veränderungsprozessen

1. Welche Qualifizierungsmaßnahmen, die die Personal- bzw. Organisationsentwicklung betreffen, haben Sie als Leitung bislang in Anspruch genommen (z.B. Fortbildung zu Personalführung, Change Management)?

VI. Allgemeine Angaben zur Patientenversorgung an Ihrem SPZ

1. Ist an Ihr SPZ eine Frühförderstelle angegliedert?

☐ Nein

☐ Ja

2. Wie viele Überweisungsscheine wurden an Ihrem SPZ im Jahr 2018 insgesamt eingelöst?

|__|__|__|__| Überweisungsscheine

3. Wie hoch ist die durchschnittliche Anzahl der Eltern-Patienten-Fachkraftkontakte pro Überweisungsschein an Ihrem SPZ?

|__|__| Kontakte

4. Gibt es an Ihrem SPZ Vorgaben bezüglich der Termindauer je nach Berufsgruppe der Fachkräfte?

☐ Nein
☐ Ja ☞ *Bitte fassen Sie diese Vorgaben im folgenden Feld kurz zusammen.*

5. Erhält Ihr SPZ neben den Mitteln der Vergütung durch die gesetzliche Krankenversicherung zusätzliche finanzielle Mittel?
(z.B. von Stiftungen, kommunalen Trägern)

☐ Nein
☐ Ja, und zwar für geschätzt |__|__| % der Patienten

6. Bitte schätzen Sie: Wie viel Prozent der Patienten, die an Ihrem SPZ betreut werden, …
☞ *Bitte machen Sie in jeder Zeile ein Kreuz auf der Skala.*

	0%	10%	20%	30%	40%	50%	60%	70%	80%	90%	100%
… sind über ihre Eltern privat versichert?											
… sind über ihre Eltern bei der AOK versichert?											
… sind über ihre Eltern bei der TK versichert?											
… sind über ihre Eltern bei der DAK versichert?											
… werden mit ihren Familien über das Jugendamt mitbetreut?											

7. Bitte schätzen Sie: Wie viel Prozent der Patienten, die an Ihrem SPZ betreut werden, …
☞ *Bitte machen Sie in jeder Zeile ein Kreuz auf der Skala.*

	0%	10%	20%	30%	40%	50%	60%	70%	80%	90%	100%
… sind zwischen 0 und 2 Jahre alt?											
… sind 7 Jahre alt oder älter?											
… sind körperlich zu schwer beeinträchtigt, um einen einfachen Fragebogen beantworten zu können?											
… sind geistig zu schwer beeinträchtigt, um einen einfachen Fragebogen beantworten zu können?											
… sind sowohl körperlich als auch geistig zu schwer beeinträchtigt, um einen											

einfachen Fragebogen beantworten zu können?	
… werden voraussichtlich nicht über die nächsten 2 Jahre regelmäßig am SPZ betreut?	**0%** 10% 20% 30% 40% **50%** 60% 70% 80% 90% **100%**

8. Bitte schätzen Sie: Wie viel Prozent der Eltern, deren Kinder an Ihrem SPZ betreut werden,…

☞ *Bitte machen Sie in jeder Zeile ein Kreuz auf der Skala.*

… sprechen nicht ausreichend deutsch, um einen einfachen Fragebogen beantworten zu können?	**0%** 10% 20% 30% 40% **50%** 60% 70% 80% 90% **100%**
… benötigen zusätzliche externe soziale und familiäre Unterstützung (z.B. Erziehungs-beratung, Frühe Hilfen, Teilnahme an speziellen sozialen Programmen außerhalb des Jugendamtes wie Müttercafé, Sprachprogramme, Patenschaften)	
… haben eine psychiatrische Erkrankung (z.B. Suchterkrankung)?	**0%** 10% 20% 30% 40% **50%** 60% 70% 80% 90% **100%**

9. Wie effizient schätzen Sie derzeit …

☞ *Bitte machen Sie in jeder Zeile ein Kreuz.*

	**Überhaupt nicht effizient**									**Sehr effizient**
	0	1	2	3	4	5	6	7	8	9
… die konkrete Arbeit mit Patienten und Eltern in Ihrem SPZ ein?	☐	☐	☐	☐	☐	☐	☐	☐	☐	☐
… die Therapieplanung (inkl. Vorbereitung und Terminvereinbarung) ein?	☐	☐	☐	☐	☐	☐	☐	☐	☐	☐
… die Dokumentation in Ihrem SPZ ein?	☐	☐	☐	☐	☐	☐	☐	☐	☐	☐
… die ***fallbezogene*** interdisziplinäre Zusammenarbeit in Ihrem SPZ ein?	☐	☐	☐	☐	☐	☐	☐	☐	☐	☐
… die ***fallunabhängige*** interdisziplinäre Zusammenarbeit in Ihrem SPZ (z.B. Erweiterung von Therapiestandards, Standards in Betrachtung von Krankheitsbildern) ein?	☐	☐	☐	☐	☐	☐	☐	☐	☐	☐
… die ***einrichtungsübergreifende*** Zusammenarbeit (z.B.Kinderärzte, Jugend-/Sozialamt, Frühförderstellen) ein?	☐	☐	☐	☐	☐	☐	☐	☐	☐	☐

VII. Angaben zu Ihrer Person

1. Ihr Geschlecht

☐ Männlich ☐ Weiblich

2. Welche Fachrichtung haben Sie?

☐ Kinderheilkunde ☐ andere Fachrichtung:____

3. Welche (Zusatz-)Weiterbildungen haben Sie absolviert?

____

4. Ihre Erfahrung in Ihrem <u>derzeitigen</u> Fachbereich: |__|__| Jahre

5. Seit wann leiten Sie dieses SPZ? Seit: |__|__|__|__|

6. Wie hoch ist Ihre derzeitige durchschnittliche Gesamtarbeitszeit pro Woche (inklusive evtl. anfallender Überstunden)? |__|__|__| Stunden

7. Wenn Sie an Ihre durchschnittliche wöchentliche Arbeitszeit am SPZ denken (inkl. anfallender Überstunden): Wie viele Stunden wenden Sie gegenwärtig für folgende leitungsbezogene Aufgaben auf?

Für die Weiterentwicklung Ihres Teams |__|__| Stunden pro Woche
Für die Weiterentwicklung der Organisationsstruktur am SPZ |__|__| Stunden pro Woche
Für administrative Aufgaben (z.B. Unterzeichnung von Verträgen) |__|__| Stunden pro Woche

8. Haben Sie die ICF-CY in der Vergangenheit bereits angewendet?

☐ Nein
☐ Ich habe von der ICF-CY schon einmal gehört
☐ Ich habe die ICF-CY schon einmal angewendet
☐ Ich wende die ICF-CY gelegentlich an
☐ Ich wende die ICF-CY regelmäßig an
☐ Ich wende die ICF-CY immer an

☞ *Bitte beschreiben Sie hier kurz Ihre bisherigen Erfahrungen mit der ICF-CY*

Zum Schluss geben Sie bitte Ihren Code an, den Sie gewählt haben

Zur Erinnerung:

In die ersten zwei Kästchen notieren Sie die **zwei letzten Buchstaben des ersten Vornamens Ihrer Mutter**.
In die nächsten zwei Kästchen notieren Sie die **zwei letzten Buchstaben des Nachnamens Ihrer Mutter**.
In die folgenden zwei Kästchen notieren **Sie den Geburtstag Ihrer Mutter**.
In die letzten zwei Kästchen notieren Sie **den Geburtsmonat Ihrer Mutter**.

Beispiel: Ihre Mutter heißt Pet**<u>ra</u>** Johanna Musterfr**<u>au</u>**. Ihre Mutter hat am **09.04.** Geburtstag.

Der Code lautet daher:

R	A	A	U	0	9	0	4

Vielen Dank für Ihre Teilnahme!

Bitte geben Sie den ausgefüllten Fragebogen **in den beiliegenden Umschlag, verschließen Sie diesen** und schicken Sie ihn zusammen mit den Fragebögen Ihrer Mitarbeiter an das Projektteam. Ihre Daten werden nur von den Projektmitarbeitern weiterverarbeitet. Die Auswertung erfolgt aggregiert, sodass keine Rückschlüsse auf Ihre Person möglich sind.

Fachkräfte-Befragung (post-interventionell)

Bitte geben Sie den ausgefüllten Fragebogen **in den beiliegenden Umschlag, verschließen Sie diesen und legen Sie ihn in die dafür vorgesehene Kiste in Ihrem SPZ-Sekretariat.** Die Fragebögen werden dann gesammelt an das Projektteam weitergeleitet. Ihre Daten werden nur von den Projektmitarbeitern weiterverarbeitet. Die Auswertung erfolgt aggregiert, sodass keine Rückschlüsse auf Ihre Person möglich sind.

Dieser Fragebogen wird ausgefüllt am: |__|__|.|__|__|.| 2 | 0 |__|__|
Tag Monat Jahr

Machen Sie bitte zu jeder Frage eine Angabe, auch wenn Ihnen manche Fragen bekannt vorkommen. Für die Prozessevaluation von PART-CHILD möchten wir gewisse Aspekte im Verlauf beurteilen.

Haben Sie an der letzten Befragung teilgenommen und den Fragebogen ausgefüllt? ☐ ja ☐ nein

I. Patienten- und Elternzentrierung im täglichen Arbeiten

1. Bitte geben Sie an, **inwiefern folgende Aspekte** bei Ihrer Arbeit mit Patienten bzw. Eltern für Sie **wichtig sind**.

☞ *Bitte machen Sie in* <u>*jeder*</u> *Zeile* <u>*ein*</u> *Kreuz. Wenn Sie als Fachkraft nur mit Kleinkindern bzw. mit schwerbehinderten Patienten arbeiten, kreuzen Sie bitte „nicht zutreffend" an.*

Patienten	**Überhaupt nicht wichtig** 0	1	2	3	4	5	6	7	8	**Sehr wichtig** 9	**Nicht zutreffend**
… bekommen stets klare Informationen.	☐	☐	☐	☐	☐	☐	☐	☐	☐	☐	☐
… bekommen immer Gelegenheit Fragen zu stellen.	☐	☐	☐	☐	☐	☐	☐	☐	☐	☐	☐
… äußern ihre Meinungen.	☐	☐	☐	☐	☐	☐	☐	☐	☐	☐	☐
… werden stets in die Besprechung von Versorgungs- bzw. Therapieoptionen einbezogen.	☐	☐	☐	☐	☐	☐	☐	☐	☐	☐	☐
… werden gleichberechtigt an Entscheidungen über ihre Versorgung bzw. Therapie beteiligt.	☐	☐	☐	☐	☐	☐	☐	☐	☐	☐	☐
… übernehmen die Verantwortung für ihre Gesundheit.	☐	☐	☐	☐	☐	☐	☐	☐	☐	☐	☐

Eltern	**Überhaupt nicht wichtig** 0	1	2	3	4	5	6	7	8	**Sehr wichtig** 9
… bekommen stets klare Informationen.	☐	☐	☐	☐	☐	☐	☐	☐	☐	☐
… bekommen immer Gelegenheit Fragen zu stellen.	☐	☐	☐	☐	☐	☐	☐	☐	☐	☐
… äußern ihre Meinungen.	☐	☐	☐	☐	☐	☐	☐	☐	☐	☐
… werden stets in die Besprechung von Versorgungs- bzw. Therapieoptionen einbezogen.	☐	☐	☐	☐	☐	☐	☐	☐	☐	☐
… werden gleichberechtigt an Entscheidungen über ihre Versorgung bzw. Therapie ihrer Kinder beteiligt.	☐	☐	☐	☐	☐	☐	☐	☐	☐	☐
… übernehmen die Verantwortung für die Gesundheit ihrer Kinder.	☐	☐	☐	☐	☐	☐	☐	☐	☐	☐

2. Bitte geben Sie an, **wie gut Sie in der Regel folgende Aspekte** bei Ihrer Arbeit mit Patienten bzw. Eltern **umsetzen können**.

☞ *Bitte machen Sie in jeder Zeile ein Kreuz. Wenn Sie als Fachkraft nur mit Kleinkindern bzw. mit schwerbehinderten Patienten arbeiten, kreuzen Sie bitte „nicht zutreffend“ an.*

Patienten	Überhaupt nicht gut 0	1	2	3	4	5	6	7	8	Sehr gut 9	Nicht zutreffend
… bekommen stets klare Informationen.	☐	☐	☐	☐	☐	☐	☐	☐	☐	☐	☐
… bekommen immer Gelegenheit Fragen zu stellen.	☐	☐	☐	☐	☐	☐	☐	☐	☐	☐	☐
… äußern ihre Meinungen.	☐	☐	☐	☐	☐	☐	☐	☐	☐	☐	☐
… werden stets in die Besprechung von Versorgungs- bzw. Therapieoptionen einbezogen.	☐	☐	☐	☐	☐	☐	☐	☐	☐	☐	☐
… werden gleichberechtigt an Entscheidungen über ihre Versorgung bzw. Therapie beteiligt.	☐	☐	☐	☐	☐	☐	☐	☐	☐	☐	☐
… übernehmen die Verantwortung für ihre Gesundheit.	☐	☐	☐	☐	☐	☐	☐	☐	☐	☐	☐

Eltern	Überhaupt nicht gut 0	1	2	3	4	5	6	7	8	Sehr gut 9
… bekommen stets klare Informationen.	☐	☐	☐	☐	☐	☐	☐	☐	☐	☐
… bekommen immer Gelegenheit Fragen zu stellen.	☐	☐	☐	☐	☐	☐	☐	☐	☐	☐
… äußern ihre Meinungen.	☐	☐	☐	☐	☐	☐	☐	☐	☐	☐
… werden stets in die Besprechung von Versorgungs- bzw. Therapieoptionen einbezogen.	☐	☐	☐	☐	☐	☐	☐	☐	☐	☐
… werden gleichberechtigt an Entscheidungen über ihre Versorgung bzw. Therapie ihrer Kinder beteiligt.	☐	☐	☐	☐	☐	☐	☐	☐	☐	☐
… übernehmen die Verantwortung für die Gesundheit ihrer Kinder.	☐	☐	☐	☐	☐	☐	☐	☐	☐	☐

II. Umsetzung der Schulungsinhalte im SPZ-Alltag

1. Nach der PART-CHILD-Teamschulung: Wie häufig wenden Sie folgende Aspekte in Ihrer Arbeit mit Patienten und Eltern an?

☞ *Bitte machen Sie in jeder Zeile ein Kreuz.*

	wende ich regelmäßig an	wende ich gelegentlich an	habe ich schon einmal angewendet	habe ich noch nie angewendet
Ich richte mein diagnostisches und beratendes Tun an Alltagsthemen des Patienten aus.	☐	☐	☐	☐
Ich erfasse das, was der Patient im Alltag macht, indem ich den Teilhabestatus (LAKMoSHIBeG) oder Teile davon mit Eltern und wenn möglich auch mit dem Patienten erhebe.	☐	☐	☐	☐
Ich erarbeite die Teilhabepräferenz des Patienten mit den Eltern und wenn möglich auch mit dem Patienten gemeinsam.	☐	☐	☐	☐
Ich ordne die wichtigsten Aspekte bei der Erstellung des Briefes alleine oder mit anderen Fachkräften den ICF-Komponenten zu (Erstellung des ICF-Profils).	☐	☐	☐	☐
Ich vergebe für die wichtigsten Aspekte bei der Erstellung der Briefe ICF-Codes und/oder ICF-Schlüsselworte.	☐	☐	☐	☐
Ich verwende die ICF-Web-App für die Erstellung der Briefe.	☐	☐	☐	☐
Ich ordne die wichtigsten Aspekte in einem gemeinsamen Gespräch (meistens nach Abschluss der Diagnostik im SPZ) mit Eltern, wenn möglich mit dem Patienten und ggf. weiteren Fachkräften den ICF-Komponenten zu (Erstellung des ICF-Profils).	☐	☐	☐	☐
Ich erarbeite Teilhabeziele gemeinsam mit Eltern und wenn möglich auch mit dem Patienten, indem ich sie durch meine Gesprächsführung auf dem Weg vom Wunsch zum Ziel begleite.	☐	☐	☐	☐
Ich richte mein therapeutisches Tun bzw. meine Therapieplanung an dem Teilhabeziel des Patienten (ggf. von Eltern oder anderen für den Patienten formuliert) aus.	☐	☐	☐	☐
Ich erarbeite mit Eltern, wenn möglich mit dem Patienten und ggf. weiteren Fachkräften gemeinsam auf dem Boden der Teilhabeziele und ICF-Profile einen Handlungsplan.	☐	☐	☐	☐
Ich beteilige Eltern an Entscheidungsprozessen.	☐	☐	☐	☐
Ich beteilige Patienten (ca. > 5 Jahre kognitives Niveau) an Entscheidungsprozessen.	☐	☐	☐	☐

2. Verwenden Sie die ICF-CY Schlüsselwortliste in Ihrer alltäglichen Arbeit?

☐ nein ☞ *Bitte machen Sie mit **Frage 5** weiter*

☐ ja ☞ *Bitte machen Sie mit **Frage 3** weiter*

3. Seit welchem Zeitpunkt nach der Schulug arbeiten Sie mit der ICF-CY Schlüsselwortliste?

Ihr erster Schulungstermin im Rahmen von PART-CHILD war im: _____ (Monat) _________ (Jahr)

Ungefährer Zeitpunkt, seit dem Sie die ICF-CY Schlüsselwortliste verwenden: _____ (Monat) _________ (Jahr)

4. Bitte schätzen Sie: In wie vielen Patientenkontakten haben Sie die ICF-CY Schlüsselwortliste seither schon genutzt?

Ungefährer Anteil der Patientenkontakte in %: __________

5. Welche Auswirkungen hat das Arbeiten nach ICF oder der Einsatz der entsprechenden ICF-basierten Assessment-Hilfen bereits jetzt auf:

☞ *Bitte machen Sie in jeder Zeile ein Kreuz.*

	sehr negativ	eher negativ	keine Auswirkung	eher positiv	sehr positiv
die Diagnostik	☐	☐	☐	☐	☐
die Therapieplanung	☐	☐	☐	☐	☐
die Patientenorientierung	☐	☐	☐	☐	☐
die interdisziplinäre Kommunikation	☐	☐	☐	☐	☐
die interdisziplinäre Zusammenarbeit	☐	☐	☐	☐	☐
die einrichtungsübergreifende Zusammenarbeit	☐	☐	☐	☐	☐
die Gesprächsführung mit den Eltern und Patienten	☐	☐	☐	☐	☐
den Therapieprozess insgesamt	☐	☐	☐	☐	☐

6. Wenn die ersten Hürden beim Arbeiten nach ICF erst einmal überwunden sind: Welches Potenzial kann das Arbeiten nach ICF Ihrer Einschätzung nach in Zukunft haben für:

☞ *Bitte machen Sie in jeder Zeile ein Kreuz.*

	sehr geringes	eher geringes	kein Potenzial	eher großes	sehr großes
die Diagnostik	☐	☐	☐	☐	☐
die Therapieplanung	☐	☐	☐	☐	☐
die Patientenorientierung	☐	☐	☐	☐	☐
die interdisziplinäre Kommunikation	☐	☐	☐	☐	☐
die interdisziplinäre Zusammenarbeit	☐	☐	☐	☐	☐
die einrichtungsübergreifende Zusammenarbeit	☐	☐	☐	☐	☐
die Gesprächsführung mit den Eltern und Patienten	☐	☐	☐	☐	☐

	sehr geringes	eher geringes	kein Potenzial	eher großes	sehr großes
den Therapieprozess insgesamt	☐	☐	☐	☐	☐

7. Wie beurteilen Sie den <u>derzeitigen</u> praktischen und zeitlichen Aufwand der Arbeit nach ICF für das SPZ-Team?

kein Aufwand	geringer Aufwand	mäßiger Aufwand	großer Aufwand	sehr großer Aufwand
☐	☐	☐	☐	☐

8. Inwieweit stimmen Sie der folgenden Aussage zu:

	stimme überhaupt nicht zu	stimme eher nicht zu	weder-noch	stimme eher zu	stimme voll und ganz zu
Wenn das Arbeiten nach ICF in Ihrem SPZ zur Routine geworden ist, steht der zu diesem Zeitpunkt zu erwartende Nutzen in einem angemessenen Verhältnis zum Aufwand.	☐	☐	☐	☐	☐

9. Welche Barrieren erschweren Ihnen persönlich das Arbeiten nach ICF in Ihrem beruflichen Alltag?

☞ *Bitte machen Sie in <u>jeder</u> Zeile <u>ein</u> Kreuz.*

	keine Barriere	geringe Barriere	mäßige Barriere	starke Barriere	sehr starke Barriere
Barrieren der SPZ-Struktur *(z.B. keine Zeit, nicht passende Abläufe innerhalb des SPZ, Teamgröße)*	☐	☐	☐	☐	☐
Sonstige strukturelle Barrieren, und zwar: ______________________________	☐	☐	☐	☐	☐
Barrieren der Versorgungsstruktur *(z.B. Finanzierung über Pauschale, Vorgaben vom SPZ-Träger)*	☐	☐	☐	☐	☐
Inhaltliche Faktoren *(z.B. Konzepte der ICF-CY, Vereinbarkeit mit schon bestehenden Klassifikationen im SPZ)*	☐	☐	☐	☐	☐
Sonstiges, und zwar: ______________________________	☐	☐	☐	☐	☐

10. Haben Sie nach der PART-CHILD-Teamschulung an weiteren <u>externen</u> Fortbildungen zu einzelnen vorgestellten Schulungsinhalten teilgenommen? Wenn ja, zu welchen Themen? (z.B. ICF-CY, Motivational Interviewing)

☐ Ja, und zwar: ____

☐ Nein

11. Wie groß sind Ihrer Einschätzung nach die Auswirkungen der Corona-Pandemie auf die Umsetzung der Schulungsinhalte in Ihrem Arbeitsalltag?

sehr gering	eher gering	mittel	eher groß	sehr groß
☐	☐	☐	☐	☐

III. Allgemeine Angaben zur Patientenversorgung an Ihrem SPZ

1. Wie viele Patientenkontakte haben Sie derzeit durchschnittlich pro Woche?

Präsenztermine: |__|__|__|, davon dauern: |__|__|__| über 2 Stunden
|__|__|__| 1 bis 2 Stunden
|__|__|__| unter 1 Stunde

Telefonische/ E-Mail-Kontakte: |__|__|__|

2. Wie hoch ist Ihre derzeitige durchschnittliche Gesamtarbeitszeit pro Woche (inklusive evtl. anfallender Überstunden)?

|__|__| Stunden

3. Wenn Sie an Ihre durchschnittliche wöchentliche Arbeitszeit im SPZ denken (inklusive evtl. anfallender Überstunden): Wie viele Stunden wenden Sie gegenwärtig für folgende Tätigkeiten auf:

Die konkrete Arbeit mit Patienten und Eltern?	______ Stunden
Die Therapieplanung (inkl. Vorbereitung und Terminvereinbarung)?	______ Stunden
Die Dokumentation (z.B. Diagnosen, Befunde, Arztberichte, Bescheinigungen)?	______ Stunden
Die fallbezogene interdisziplinäre Zusammenarbeit im SPZ (z.B. Fallkonferenzen, runde Tische)?	______ Stunden
Die einrichtungsübergreifende Zusammenarbeit (z.B. mit Jugend-/ Sozialamt)?	______ Stunden
Die Weiterentwicklung der Versorgung am SPZ (z.B. Arbeitsgruppe zur Umsetzung neuer Standards)?	______ Stunden
sonstige Tätigkeiten, z.B.: ______________________________	______ Stunden
______________________________	______ Stunden
______________________________	______ Stunden

4. Wie effizient schätzen Sie derzeit …

☞ *Bitte machen Sie in* ***jeder*** *Zeile* ***ein*** *Kreuz auf der Skala.*

	Überhaupt nicht effizient 0	1	2	3	4	5	6	7	8	Sehr effizient 9	Kann nicht beurteilen
… die konkrete Arbeit mit Patienten und Eltern in Ihrem SPZ ein?	☐	☐	☐	☐	☐	☐	☐	☐	☐	☐	☐
… die Therapieplanung (inkl. Vorbereitung und Terminvereinbarung) ein?	☐	☐	☐	☐	☐	☐	☐	☐	☐	☐	☐
… die Dokumentation in Ihrem SPZ ein?	☐	☐	☐	☐	☐	☐	☐	☐	☐	☐	☐
… die ***fallbezogene*** interdisziplinäre Zusammenarbeit in Ihrem SPZ ein?	☐	☐	☐	☐	☐	☐	☐	☐	☐	☐	☐
… die ***fallunabhängige*** interdisziplinäre Zusammenarbeit in Ihrem SPZ (z.B. Erweiterung von Therapiestandards, Standards in der Betrachtung von Krankheitsbildern) ein?	☐	☐	☐	☐	☐	☐	☐	☐	☐	☐	☐
… die ***einrichtungsübergreifende*** Zusammenarbeit (z.B.Kinderärzte, Jugend-/Sozialamt, Frühförder-stellen) ein?	☐	☐	☐	☐	☐	☐	☐	☐	☐	☐	☐

5. Bitte denken Sie an das letzte Quartal: Wie hoch war der Anteil Ihrer Patienten und Eltern, die sich ziemlich genau an getroffene Vereinbarungen gehalten haben bezüglich der…

☞ *Bitte machen Sie in* ***jeder*** *Zeile* ***ein*** *Kreuz auf der Skala.*

Wenn ein Punkt für Ihre Berufsgruppe nicht beurteilbar ist, kreuzen Sie „Kann nicht beurteilen“ an.

	0%	10%	20%	30%	40%	50%	60%	70%	80%	90%	100%	Kann nicht beurteilen
Einnahme verordneter Medikamente?												☐
Inanspruchnahme von sonstigen nicht-ärztlichen Leistungen?												
Ergotherapie												☐
Logopädie												☐
Physiotherapie												☐
Sonstige Therapien: ______________________________												☐
Ausstattung mit Hilfsmitteln (z.B. Gehhilfen, Schreibhilfen, Rollstuhl)?												☐
Inanspruchnahme von Schulungen bzw. Beratungen (z.B. Elterntraining, Beratung zur Erziehung, sozialen Hilfen)?												☐

IV. Allgemeine Zufriedenheit mit der beruflichen Tätigkeit

☞ *Bitte machen Sie in jeder Zeile ein Kreuz.*

	Stimme überhaupt nicht zu 0	1	2	3	4	5	6	7	8	Stimme voll und ganz zu 9
Ich persönlich empfinde meine derzeitige Arbeit im SPZ als lohnend.	☐	☐	☐	☐	☐	☐	☐	☐	☐	☐
Insgesamt gefällt mir meine Arbeit hier im SPZ.	☐	☐	☐	☐	☐	☐	☐	☐	☐	☐
Insgesamt bin ich mit meiner derzeitigen Arbeit zufrieden.	☐	☐	☐	☐	☐	☐	☐	☐	☐	☐
Meine derzeitige Arbeitssituation bietet für mich großen Anlass zur Frustration.	☐	☐	☐	☐	☐	☐	☐	☐	☐	☐
Meine Arbeit im SPZ entspricht nicht meinen Erwartungen.	☐	☐	☐	☐	☐	☐	☐	☐	☐	☐
Ich empfinde meine derzeitige Arbeit im SPZ als sinnstiftend.	☐	☐	☐	☐	☐	☐	☐	☐	☐	☐
Meine derzeitige Arbeitssituation ist durch Zeitdruck geprägt.	☐	☐	☐	☐	☐	☐	☐	☐	☐	☐
Meine derzeitige Arbeitssituation ist durch Kostendruck geprägt.	☐	☐	☐	☐	☐	☐	☐	☐	☐	☐
In meiner Arbeit kann ich viele Entscheidungen selbst treffen.	☐	☐	☐	☐	☐	☐	☐	☐	☐	☐
Ich habe sehr wenig Entscheidungsspielraum darüber, wie ich meine Arbeit erledige.	☐	☐	☐	☐	☐	☐	☐	☐	☐	☐

Zum Schluss geben Sie bitte Ihren Code an, den Sie gewählt haben

Zur Erinnerung:

In die ersten zwei Kästchen notieren Sie die **zwei letzten Buchstaben des ersten Vornamens Ihrer Mutter**.
In die nächsten zwei Kästchen notieren Sie die **zwei letzten Buchstaben des Nachnamens Ihrer Mutter**.
In die folgenden zwei Kästchen notieren **Sie den Geburtstag Ihrer Mutter**.
In die letzten zwei Kästchen notieren Sie **den Geburtsmonat Ihrer Mutter**.

Beispiel: Ihre Mutter heißt Pet**ra** Johanna Musterfr**au**. Ihre Mutter hat am **09.04.** Geburtstag.

Der Code lautet daher:

R	A	A	U	0	9	0	4

Vielen Dank für Ihre Teilnahme!

Bitte geben Sie den ausgefüllten Fragebogen **in den beiliegenden Umschlag, verschließen Sie diesen und legen Sie ihn in die dafür vorgesehene Kiste in Ihrem SPZ-Sekretariat.** Die Fragebögen werden dann gesammelt an das Projektteam weitergeleitet. Ihre Daten werden nur von den Projektmitarbeitern weiterverarbeitet. Die Auswertung erfolgt aggregiert, sodass keine Rückschlüsse auf Ihre Person möglich sind.

Befragung der SPZ-Leitung *(post-interventionell)*

Bitte geben Sie den ausgefüllten Fragebogen **in den beiliegenden Umschlag, verschließen Sie diesen** und schicken Sie ihn zusammen mit den Fragebögen Ihrer Mitarbeiter an das Projektteam. Ihre Daten werden nur von den Projektmitarbeitern weiterverarbeitet. Die Auswertung erfolgt aggregiert, sodass keine Rückschlüsse auf Ihre Person möglich sind.

Dieser Fragebogen wird ausgefüllt am: |__|__|.|__|__|.| 2 | 0 |__|__|
Tag Monat Jahr

Machen Sie bitte zu jeder Frage eine Angabe, auch wenn Ihnen manche Fragen bekannt vorkommen. Für die Prozessevaluation von PART-CHILD möchten wir gewisse Aspekte im Verlauf beurteilen.

I. Patienten- und Elternzentrierung im täglichen Arbeiten

1. Bitte geben Sie an, **inwiefern folgende Aspekte** in Ihrer Arbeit mit Patienten bzw. Eltern für Sie **wichtig sind**.
☞ *Bitte machen Sie in jeder Zeile ein Kreuz. Wenn Sie als Fachkraft nur mit Kleinkindern bzw. mit schwerbehinderten Patienten arbeiten, kreuzen Sie bitte „nicht zutreffend" an.*

Patienten	**Überhaupt nicht wichtig** 0	1	2	3	4	5	6	7	8	**Sehr wichtig** 9	**Nicht zutreffend**
… bekommen stets klare Informationen.	☐	☐	☐	☐	☐	☐	☐	☐	☐	☐	☐
… bekommen immer Gelegenheit, Fragen zu stellen.	☐	☐	☐	☐	☐	☐	☐	☐	☐	☐	☐
… äußern ihre Meinungen.	☐	☐	☐	☐	☐	☐	☐	☐	☐	☐	☐
… werden stets in die Besprechung von Versorgungs- bzw. Therapieoptionen einbezogen.	☐	☐	☐	☐	☐	☐	☐	☐	☐	☐	☐
… werden gleichberechtigt an Entscheidungen über ihre Versorgung bzw. Therapie beteiligt.	☐	☐	☐	☐	☐	☐	☐	☐	☐	☐	☐
… übernehmen die Verantwortung für ihre Gesundheit.	☐	☐	☐	☐	☐	☐	☐	☐	☐	☐	☐

Eltern	**Überhaupt nicht wichtig** 0	1	2	3	4	5	6	7	8	**Sehr wichtig** 9
… bekommen stets klare Informationen.	☐	☐	☐	☐	☐	☐	☐	☐	☐	☐
… bekommen immer Gelegenheit, Fragen zu stellen.	☐	☐	☐	☐	☐	☐	☐	☐	☐	☐
… äußern ihre Meinungen.	☐	☐	☐	☐	☐	☐	☐	☐	☐	☐
… werden stets in die Besprechung von Versorgungs- bzw. Therapieoptionen einbezogen.	☐	☐	☐	☐	☐	☐	☐	☐	☐	☐
… werden gleichberechtigt an Entscheidungen über ihre Versorgung bzw. Therapie ihrer Kinder beteiligt.	☐	☐	☐	☐	☐	☐	☐	☐	☐	☐
… übernehmen die Verantwortung für die Gesundheit ihrer Kinder.	☐	☐	☐	☐	☐	☐	☐	☐	☐	☐

2. Bitte geben Sie an, **wie gut Sie in der Regel folgende Aspekte** in Ihrer Arbeit mit Patienten bzw. Eltern **umsetzen können**.

☞ *Bitte machen Sie in* *jeder* *Zeile* *ein* *Kreuz. Wenn Sie als Fachkraft nur mit Kleinkindern bzw. mit schwerbehinderten Patienten arbeiten, kreuzen Sie bitte „nicht zutreffend" an.*

Patienten	**Überhaupt nicht gut** 0	1	2	3	4	5	6	7	8	**Sehr gut** 9	**Nicht zutreffend**
… bekommen stets klare Informationen.	☐	☐	☐	☐	☐	☐	☐	☐	☐	☐	☐
… bekommen immer Gelegenheit Fragen zu stellen.	☐	☐	☐	☐	☐	☐	☐	☐	☐	☐	☐
… äußern ihre Meinungen.	☐	☐	☐	☐	☐	☐	☐	☐	☐	☐	☐
… werden stets in die Besprechung von Versorgungs- bzw. Therapieoptionen einbezogen.	☐	☐	☐	☐	☐	☐	☐	☐	☐	☐	☐
… werden gleichberechtigt an Entscheidungen über ihre Versorgung bzw. Therapie beteiligt.	☐	☐	☐	☐	☐	☐	☐	☐	☐	☐	☐
… übernehmen die Verantwortung für ihre Gesundheit.	☐	☐	☐	☐	☐	☐	☐	☐	☐	☐	☐

Eltern	**Überhaupt nicht gut** 0	1	2	3	4	5	6	7	8	**Sehr gut** 9
… bekommen stets klare Informationen.	☐	☐	☐	☐	☐	☐	☐	☐	☐	☐
… bekommen immer Gelegenheit Fragen zu stellen.	☐	☐	☐	☐	☐	☐	☐	☐	☐	☐
… äußern ihre Meinungen.	☐	☐	☐	☐	☐	☐	☐	☐	☐	☐
… werden stets in die Besprechung von Versorgungs- bzw. Therapieoptionen einbezogen.	☐	☐	☐	☐	☐	☐	☐	☐	☐	☐
… werden gleichberechtigt an Entscheidungen über die Versorgung bzw. Therapie ihrer Kinder beteiligt.	☐	☐	☐	☐	☐	☐	☐	☐	☐	☐
… übernehmen die Verantwortung für die Gesundheit ihrer Kinder.	☐	☐	☐	☐	☐	☐	☐	☐	☐	☐

II. Umsetzung der Schulungsinhalte im SPZ-Alltag

1. Welche Aktivitäten wurden in Ihrem SPZ nach der PART-CHILD-Teamschulung ins Leben gerufen, um die Umsetzung einzelner Schulungsinhalte in der täglichen Arbeit zu unterstützen?

2. Wurden nach der PART-CHILD-Teamschulung Schulungsinhalte in SPZ-internen Fortbildungen außerhalb der Impelemtierungsbegleitung vertieft?
Wenn ja, zu welchen Themen fanden SPZ-interne Fortbildungen statt? (z.B. ICF-CY, Motivational Interviewing)

☐ Ja, und zwar: ____________________

☐ Nein

3. Nach der PART-CHILD-Teamschulung: Wie häufig wenden Sie folgende Aspekte in Ihrer Arbeit mit Patienten und Eltern an?

☞ *Bitte machen Sie in jeder Zeile ein Kreuz.*

	**wende ich regelmäßig an**	**wende ich gelegentlich an**	**habe ich schon einmal angewendet**	**habe ich noch nie angewendet**
Ich richte mein diagnostisches und beratendes Tun an Alltagsthemen des Patienten aus.	☐	☐	☐	☐
Ich erfasse das, was der Patient im Alltag macht, indem ich den Teilhabestatus (LAKMoSHIBeG) oder Teile davon mit Eltern und wenn möglich auch mit dem Patienten erhebe.	☐	☐	☐	☐
Ich erarbeite die Teilhabepräferenz des Patienten mit den Eltern und wenn möglich auch mit dem Patienten gemeinsam.	☐	☐	☐	☐
Ich ordne die wichtigsten Aspekte bei der Erstellung des Briefes alleine oder mit anderen Fachkräften den ICF-Komponenten zu (Erstellung des ICF-Profils).	☐	☐	☐	☐
Ich vergebe für die wichtigsten Aspekte bei der Erstellung der Briefe ICF-Codes und/oder ICF-Schlüsselworte.	☐	☐	☐	☐
Ich verwende die ICF-Web-App für die Erstellung der Briefe.	☐	☐	☐	☐
Ich ordne die wichtigsten Aspekte in einem gemeinsamen Gespräch (meistens nach Abschluss der Diagnostik im SPZ) mit Eltern, wenn möglich mit dem Patienten und ggf. weiteren Fachkräften den ICF-Komponenten zu (Erstellung des ICF-Profils).	☐	☐	☐	☐
Ich erarbeite Teilhabeziele gemeinsam mit Eltern und wenn möglich auch mit dem Patienten, indem ich sie durch meine Gesprächsführung auf dem Weg vom Wunsch zum Ziel begleite.	☐	☐	☐	☐
Ich richte mein therapeutisches Tun bzw. meine Therapieplanung an dem Teilhabeziel des Patienten (ggf. von Eltern oder anderen für den Patienten formuliert) aus.	☐	☐	☐	☐
Ich erarbeite mit Eltern, wenn möglich mit dem Patienten und ggf. weiteren Fachkräften gemeinsam auf dem Boden der Teilhabeziele und ICF-Profile einen Handlungsplan.	☐	☐	☐	☐
Ich beteilige Eltern an Entscheidungsprozessen.	☐	☐	☐	☐
Ich beteilige Patienten (ca. > 5 Jahre kognitives Niveau) an Entscheidungsprozessen.	☐	☐	☐	☐

4. Verwenden Sie die ICF-CY Schlüsselwortliste in Ihrer alltäglichen Arbeit?

☐ nein ☞ *Bitte machen Sie mit **Frage 7** weiter*

☐ ja ☞ *Bitte machen Sie mit **Frage 5** weiter*

5. Seit welchem Zeitpunkt nach der Schulug arbeiten Sie mit der ICF-CY Schlüsselwortliste?

Ihr erster Schulungstermin im Rahmen von PART-CHILD war im: _____ (Monat) _________ (Jahr)

Ungefährer Zeitpunkt, seit dem Sie die ICF-CY Schlüsselwortliste verwenden: _____ (Monat) _________ (Jahr)

6. Bitte schätzen Sie: In wie vielen Patientenkontakten haben Sie die ICF-CY Schlüsselwortliste seither schon genutzt?

Ungefährer Anteil der Patientenkontakte in %: __________

7. Welche Auswirkungen hat das Arbeiten nach ICF oder der Einsatz der entsprechenden ICF-basierten Assessment-Hilfen bereits jetzt auf:

☞ *Bitte machen Sie in jeder Zeile ein Kreuz.*

	sehr negativ	eher negativ	keine Auswirkung	eher positiv	sehr positiv
die Diagnostik	☐	☐	☐	☐	☐
die Therapieplanung	☐	☐	☐	☐	☐
die Patientenorientierung	☐	☐	☐	☐	☐
die interdisziplinäre Kommunikation	☐	☐	☐	☐	☐
die interdisziplinäre Zusammenarbeit	☐	☐	☐	☐	☐
die einrichtungsübergreifende Zusammenarbeit	☐	☐	☐	☐	☐
die Gesprächsführung mit den Eltern und Patienten	☐	☐	☐	☐	☐
den Therapieprozess insgesamt	☐	☐	☐	☐	☐

8. Wenn die ersten Hürden beim Arbeiten nach ICF erst einmal überwunden sind: Welches Potenzial kann das Arbeiten nach ICF Ihrer Einschätzung nach in Zukunft haben für:

☞ *Bitte machen Sie in jeder Zeile ein Kreuz.*

	sehr geringes	eher geringes	kein Potenzial	eher großes	sehr großes
die Diagnostik	☐	☐	☐	☐	☐
die Therapieplanung	☐	☐	☐	☐	☐
die Patientenorientierung	☐	☐	☐	☐	☐
die interdisziplinäre Kommunikation	☐	☐	☐	☐	☐
die interdisziplinäre Zusammenarbeit	☐	☐	☐	☐	☐
die einrichtungsübergreifende Zusammenarbeit	☐	☐	☐	☐	☐
die Gesprächsführung mit den Eltern und Patienten	☐	☐	☐	☐	☐

	sehr geringes	eher geringes	kein Potenzial	eher großes	sehr großes
den Therapieprozess insgesamt	☐	☐	☐	☐	☐

9. Wie beurteilen Sie den <u>derzeitigen</u> praktischen und zeitlichen Aufwand der Arbeit nach ICF für das SPZ-Team?

kein Aufwand	geringer Aufwand	mäßiger Aufwand	großer Aufwand	sehr großer Aufwand
☐	☐	☐	☐	☐

10. Inwieweit stimmen Sie der folgenden Aussage zu:

	stimme überhaupt nicht zu	stimme eher nicht zu	weder-noch	stimme eher zu	stimme voll und ganz zu
Wenn das Arbeiten nach ICF in Ihrem SPZ zur Routine geworden ist, steht der zu diesem Zeitpunkt zu erwartende Nutzen in einem angemessenen Verhältnis zum Aufwand.	☐	☐	☐	☐	☐

11. Welche Barrieren erschweren Ihnen persönlich das Arbeiten nach ICF in Ihrem beruflichen Alltag?

☞ *Bitte machen Sie in <u>jeder</u> Zeile <u>ein</u> Kreuz.*

	keine Barriere	geringe Barriere	mäßige Barriere	starke Barriere	sehr starke Barriere
Barrieren der SPZ-Struktur *(z.B. keine Zeit, nicht passende Abläufe innerhalb des SPZ, Teamgröße)*	☐	☐	☐	☐	☐
Sonstige strukturelle Barrieren, und zwar: ___________________________________	☐	☐	☐	☐	☐
Barrieren der Versorgungsstruktur *(z.B. Finanzierung über Pauschale, Vorgaben vom SPZ-Träger)*	☐	☐	☐	☐	☐
Inhaltliche Faktoren *(z.B. Konzepte der ICF-CY, Vereinbarkeit mit schon bestehenden Klassifikationen im SPZ)*	☐	☐	☐	☐	☐
Sonstiges, und zwar: ___________________________________	☐	☐	☐	☐	☐

12. Inwieweit stimmen Sie den folgenden Aussagen zu:

	stimme überhaupt nicht zu 0	1	2	3	4	5	6	7	8	stimme voll und ganz zu 9
In unserem SPZ sind die ICF-Themen fester Bestandteil von Routinebesprechungen.	☐	☐	☐	☐	☐	☐	☐	☐	☐	☐
Neue Mitarbeiter werden strukturiert in die Inhalte der ICF-Schulungen eingeführt.	☐	☐	☐	☐	☐	☐	☐	☐	☐	☐

	stimme überhaupt nicht zu 0	1	2	3	4	5	6	7	8	stimme voll und ganz zu 9
Die Fachkräfte bilden interdisziplinäre Arbeitsgruppen zur bestmöglichen Erreichung der Teilhabeziele ihrer Patienten.	☐	☐	☐	☐	☐	☐	☐	☐	☐	☐
Das Arbeiten nach ICF wird als Qualitätskriterium der Versorgung nach außen kommuniziert.	☐	☐	☐	☐	☐	☐	☐	☐	☐	☐

13. Wie groß sind Ihrer Einschätzung nach die Auswirkungen der Corona-Pandemie auf die Umsetzung der Schulungsinhalte in Ihrem Arbeitsalltag?

sehr gering	eher gering	mittel	eher groß	sehr groß
☐	☐	☐	☐	☐

14. Inwiefern wirkt sich die Corona-Pandemie in Ihrem SPZ auf die Umsetzung der Schulungsinhalte aus?

☞ *Stichpunkte reichen aus.*

15. Inwiefern wirkt sich die Corona-Pandemie in Ihrem SPZ auf die Patientenversorgung allgemein aus?

☞ *Stichpunkte reichen aus.*

III. Angaben zu den Kosten der PART-CHILD-Intervention

Folgende Angaben sind wichtig für die ökonomische Evaluation der PART-CHILD-Intervention.

☞ *Bitte geben Sie im Folgenden die geschätzten Gesamtkkosten oder die geschätzte Summe der Stundenlöhne oder die geschätzte Anzahl der Arbeitsstunden aller jeweils beteiligten Mitarbeiter an.*

1. Auf welche Höhe beliefen sich die geschätzten Kosten, die im Rahmen der Teamschulungen entstanden sind?

… für die Schulungsräumlichkeiten?	____________ €
… für den technischen Support vor Ort?	____________ € oder<br>____________Arbeitsstunden
… für weitere Ausgaben, die im Rahmen der Schulung anfielen (z.B. Catering)?	____________ €
… Sonstiges, und zwar: ____	____________ € oder<br>____________Arbeitsstunden

2. Auf welche Höhe beliefen sich die Kosten, die im Rahmen der Implementierungsbegleitung entstanden sind?

Damit meinen wir alle Veranstaltungen, die im Rahmen der Implementierungsbegleitung in Ihrem SPZ stattfanden.

… für die Schulungsräumlichkeiten?	____________ €
… für den technischen Support vor Ort?	____________ € oder<br>____________Arbeitsstunden
… für weitere Ausgaben, die im Rahmen der Schulung anfielen (z.B. Catering)?	____________ €
… Sonstiges, und zwar: ____	____________ € oder<br>____________Arbeitsstunden

3. Welchen finanziellen Aufwand bedeutete die Einrichtung der ICF-Web-App?

Mit ICF-Web-App ist das Online-Tool gemeint, das Sie bei der Kodierung nach ICF-CY unterstützen soll und welches Sie im Internet-Browser nutzen können.

… für die IT-Abteilung Ihrer Einrichtung?	____________ € oder<br>____________Arbeitsstunden
… für den Datenschutzbeauftragten Ihrer Einrichtung?	____________ € oder<br>____________Arbeitsstunden
… für die PART-CHILD-Projektleitung (neben der SPZ-Leitung) in Ihrer Einrichtung?	____________ € oder<br>____________Arbeitsstunden
… für weitere Mitarbeiter Ihrer Einrichtung, und zwar: ______________________________	____________ € oder<br>____________Arbeitsstunden

IV. Allgemeine Angaben zur Patientenversorgung an Ihrem SPZ

1. Wie hoch ist derzeit die durchschnittliche Anzahl der Eltern-Patienten-Fachkraftkontakte pro Überweisungsschein an Ihrem SPZ?

|__|__| Kontakte

2. Wie effizient schätzen Sie derzeit …

☞ *Bitte machen Sie in jeder Zeile ein Kreuz.*

	Überhaupt nicht effizient 0	1	2	3	4	5	6	7	8	Sehr effizient 9
… die konkrete Arbeit mit Patienten und Eltern in Ihrem SPZ ein?	☐	☐	☐	☐	☐	☐	☐	☐	☐	☐
… die Therapieplanung (inkl. Vorbereitung und Terminvereinbarung) ein?	☐	☐	☐	☐	☐	☐	☐	☐	☐	☐
… die Dokumentation in Ihrem SPZ ein?	☐	☐	☐	☐	☐	☐	☐	☐	☐	☐
… die ***fallbezogene*** interdisziplinäre Zusammenarbeit in Ihrem SPZ ein?	☐	☐	☐	☐	☐	☐	☐	☐	☐	☐
… die ***fallunabhängige*** interdisziplinäre Zusammenarbeit in Ihrem SPZ (z.B. Erweiterung von Therapiestandards, Standards in der Betrachtung von Krankheitsbildern) ein?	☐	☐	☐	☐	☐	☐	☐	☐	☐	☐
… die ***einrichtungsübergreifende*** Zusammenarbeit (z.B.Kinderärzte, Jugend-/Sozialamt, Frühförderstellen) ein?	☐	☐	☐	☐	☐	☐	☐	☐	☐	☐

V. Merkmale des Arbeitsumfelds SPZ

1. Wie viele Fachkräfte, die diagnostisch, therapeutisch oder beratend tätig sind, sind derzeit in Ihrem SPZ angestellt?

☞ *Bitte geben Sie die Anzahl a) in Köpfen und b) in Vollzeitäquivalenten (VÄ) an, z.B. 3 Köpfe und 2,5 VÄ für 2 Vollzeitkräfte und eine 50%-Kraft.*

	**Köpfe**	**VÄ**
Diagnostisch, therapeutisch oder beratend tätige Fachkräfte	________	________

2. Wie viele Fachkräfte, die administrativ oder assistierend tätig sind, sind derzeit in Ihrem SPZ angestellt?

☞ *Bitte geben Sie die Anzahl a) in Köpfen und b) in Vollzeitäquivalenten (VÄ) an, z.B. 3 Köpfe und 2,5 VÄ für 2 Vollzeitkräfte und eine 50%-Kraft.*

	**Köpfe**	**VÄ**
Assistierend oder administrativ tätige Fachkräfte	________	________

VI. Allgemeine Zufriedenheit mit der beruflichen Tätigkeit

☞ *Bitte machen Sie in <u>jeder</u> Zeile <u>ein</u> Kreuz.*

	**Stimme überhaupt nicht zu** 0	1	2	3	4	5	6	7	8	**Stimme voll und ganz zu** 9
Ich persönlich empfinde meine derzeitige Arbeit im SPZ als lohnend.	☐	☐	☐	☐	☐	☐	☐	☐	☐	☐
Insgesamt gefällt mir meine Arbeit hier im SPZ.	☐	☐	☐	☐	☐	☐	☐	☐	☐	☐
Insgesamt bin ich mit meiner derzeitigen Arbeit zufrieden.	☐	☐	☐	☐	☐	☐	☐	☐	☐	☐
Meine derzeitige Arbeitssituation bietet für mich großen Anlass zur Frustration.	☐	☐	☐	☐	☐	☐	☐	☐	☐	☐
Meine Arbeit im SPZ entspricht nicht meinen Erwartungen.	☐	☐	☐	☐	☐	☐	☐	☐	☐	☐
Ich empfinde meine derzeitige Arbeit im SPZ als sinnstiftend.	☐	☐	☐	☐	☐	☐	☐	☐	☐	☐
Meine derzeitige Arbeitssituation ist durch Zeitdruck geprägt.	☐	☐	☐	☐	☐	☐	☐	☐	☐	☐
Meine derzeitige Arbeitssituation ist durch Kostendruck geprägt.	☐	☐	☐	☐	☐	☐	☐	☐	☐	☐
In meiner Arbeit kann ich viele Entscheidungen selbst treffen.	☐	☐	☐	☐	☐	☐	☐	☐	☐	☐
Ich habe sehr wenig Entscheidungsspielraum darüber, wie ich meine Arbeit erledige.	☐	☐	☐	☐	☐	☐	☐	☐	☐	☐

VII. Angaben zu Ihrer Person

1. Wie hoch ist Ihre derzeitige durchschnittliche Gesamtarbeitszeit pro Woche (inklusive evtl. anfallender Überstunden)? |__|__|__| Stunden

2. Wenn Sie an Ihre durchschnittliche wöchentliche Arbeitszeit am SPZ denken (inkl. anfallender Überstunden): Wie viele Stunden wenden Sie gegenwärtig für folgende leitungsbezogene Aufgaben auf?

Für die Weiterentwicklung Ihres Teams	|__|__| Stunden pro Woche
Für die Weiterentwicklung der Organisationsstruktur am SPZ	|__|__| Stunden pro Woche
Für administrative Aufgaben (z.B. Abwicklung von Stellenbesetzung)	|__|__| Stunden pro Woche

Online Supplementary File S3: Guides for semi-structured interviews with SPC directors and their staff

Leitfaden für die telefonischen Interviews mit Fachkräften

Interviewer: ______________
ID: _____________
SPZ_ID: _____________
Datum: _____________
Dauer: _____________

1. Einleitung

- Vorstellung der Interviewerin
- Erklärung zum Ablauf des Interviews, Erklärung zu spontanen Äußerungen und freier Assoziation
- Vorstellung des Interviewten (Alter, Berufsgruppe, Dauer der Beschäftigung im SPZ); Klärung der Teilnahme an der Mitarbeiterschulung.

Im Folgenden geht es um Ihre Erfahrungen im Rahmen der PART-CHILD-Intervention. Mit Intervention meinen wir sowohl die Mitarbeiterschulung (Haben Sie an dieser teilgenommen?), das ICF-Add-in/die ICF-Web-App und die Begleitung im SPZ-Alltag (Modul IMPLEMENT). Mit der Intervention meinen wir aber nicht die Datenerhebung, also nicht die Fragebögen, die Sie austeilen und auch selbst ausfüllen mussten.

2. Eingangsfragen (Warm up)

- Ganz allgemein betrachtet: Wie ist es Ihnen denn in der Zeit, in der PART-CHILD läuft, ergangen?

3. Hauptfragen

Veränderungen in der Haltung und in der subjektiven Praxis

- Was hat sich seit der PART-CHILD-Intervention in Ihrem Arbeitsalltag verändert?
    - *Allgemein positiv wie negativ: Z.B. Abläufe, Arbeitsweise, mehr Zeit für den Patienten, Dokumentationsaufwand*
    - *Hat sich bei der Aufteilung Ihrer wöchentlichen Arbeitszeit etwas verändert? Wenn ja, bitte quantifizieren lassen.*
    - *Wenn mehreres: Was war für Sie persönlich die wichtigste Veränderung?*
    - *Warum hat sich das verändert?*
    - *Gelingen: Was war wichtig, damit diese Veränderung gelingen konnte?*
    - *Barrieren: Was waren Barrieren für diese Veränderung?*
- Welche Veränderungen beobachten Sie bei Ihrer eigenen Einstellung zu Ihrer Tätigkeit?
    - *Vertiefen: Inwiefern ist diese Veränderung bedeutsam?*
    - *Gelingen: Welche Faktoren haben diese Veränderung unterstützt?*
    - *Barrieren: Was waren Barrieren für diese Veränderung?*
- Was hat sich durch die PART-CHILD-Intervention im Team Ihres SPZ verändert?
    - *Welche Fachgruppen tun sich mit dieser Veränderung besonders leicht?*
        - *Warum denken Sie ist das so?*
    - *Welche Berufsgruppen tun sich besonders schwer mit der Veränderung?*
        - *Warum, denken Sie ist das so?*

- ▪ *Wie könnte man diese Berufsgruppen in diesem Veränderungsprozess unterstützten?*
  - *Zusammenarbeit zwischen verschiedenen Disziplinen?*
  - *Kommunikation mit anderen Abteilungen der Klinik?*
- Wenn Sie an die Kommunikation in Ihrem SPZ denken: Welche Rolle spielt ICF-CY dabei?
  - *Z.B. sind ICF-Themen Gegenstand von Routinebesprechungen?*

- Welche Veränderungen beobachten Sie hinsichtlich der Zusammenarbeit mit externen Einrichtungen?
  (Beispiele nur bei Unklarheit nennen: Einrichtungen, mit welchen Sie am häufigsten zusammenarbeiten z.B. Kindergarten / Schule/ Frühförderung/ Jugendhilfe/ Gesundheitsamt)
  - *Veränderung in der Kommunikation?*
  - *Vertiefen: Inwiefern ist diese Veränderung bedeutsam?*
  - *Gelingen: Welche Faktoren haben diese Veränderung unterstützt?*
  - *Barrieren: Was sind Barrieren für diese Veränderung?*

- Welche Elemente aus der PART-CHILD-Intervention setzen Sie in Ihrem Arbeitsalltag ein?
  - *ICF-CY (z.B. Erfassung teilhaberelevanter Domänen nach Lakmoshibek, Codieren)?*
    - *Teilhabeorientierung & Patientenzentrierung: Therapieziele (Teilhabestatus/Teilhabepräferenz / Aktionsplan/Handlungsplan) werden zusammen mit Patienten nach bestimmten Kriterien festgelegt, Gespräche nach bestimmten Schemata geführt)*
  - *MI-Werkzeugkoffer? Formulierung der Ziele auf Patientenebene?*
  - *Wie regelmäßig wenden Sie diese Elemente an? Bei allen Patienten?*
  - *Wie zufrieden sind sie damit? Warum?*
  - *Veränderungen der diagnostischen/ therapeutischen Praxis?*
- Wie nutzen Sie derzeit ICF-CY?
  - *Warum ist es für Sie persönlich wichtig, ICF-CY anzuwenden?*
  - *Gelingen: Welche Faktoren sind bei der Einführung der ICF-CY förderlich?*
  - *Barrieren: Welche Barrieren gibt es bei der Nutzung der ICF-CY?*
  - *Wie wenden Ihre KollegInnen die ICF-CY an? Unterschiede zwischen Disziplinen?*

Einschätzung der Patienten-/Eltern-Einstellung

- Was schätzen Sie: Wie haben Ihre Patienten und Ihre Eltern von der Intervention in Ihrem SPZ profitiert?
  - *Veränderung im Umgang mit Patienten/ Eltern, Kommunikation?*
  - *Veränderungen bezügl. der Adhärenz der Patienten?*
  - *Was waren die Gründe für diese Veränderungen?*
  - *Wie kommen die Veränderungen bei Ihren Patienten/ Eltern an (Mitmachen oder lieber den Therapeuten entscheiden lassen)?*

Nutzung von Add-In/Web-App

- In Ihrem SPZ wurde Ihnen die Web-App zum praktischen Arbeiten nach ICF-CY zur Verfügung gestellt. Wurde sie denn in der Mitarbeiterschulung vorgestellt? Nutzen Sie die Web-App?

Wenn ja:

- Beschreiben Sie bitte, wie Sie die Web-App bisher typischerweise nutzen.

- *Täglich? Wie <u>häufig</u> im Schnitt am Tag?*
- *Bei welchen <u>Anlässen</u>? Bei welchen <u>Terminarten</u> eher?*
- *Wenn nicht bei allen Terminen: Was sind denn die <u>Gründe</u> dafür, dass Sie* die Web-App *nicht bei allen Gesprächen nutzen?*
- *<u>Zeitpunkt der Nutzung:</u> Vor, während oder nach den Patientengesprächen?*
- *Zu welchem <u>Zweck</u> nutzen Sie* die Web-App *(z.B. Dokumentation, interaktive Festlegung von Zielen mit Patienten und Eltern)?*

- Wissen Sie, wie Ihre <u>Kollegen</u> das Add-In in ihrer Arbeit einsetzen?
- Wie <u>zufrieden</u> sind Sie bisher mit der Web-App?
- Haben Sie <u>Anregungen für die Weiterentwicklung</u> der Web-App, damit es Ihre Arbeit noch besser unterstützt?

<u>Wenn nein:</u>

- <u>Barrieren (internal):</u> Was sind die <u>Gründe</u>, dass Sie die Web-App nicht nutzen?
- <u>Barrieren (external):</u> Welche <u>Schwierigkeiten</u> sehen Sie mit der Nutzung der Web-App im Arbeitsalltag verbunden?
  - *Zeitmangel, Nutzungsfreundlichkeit…?*
- Haben Sie <u>Anregungen für die Weiterentwicklung</u> der Web-App, damit sie Ihre Arbeit optimal unterstützen kann?
- Wissen Sie, ob Ihre <u>Kollegen</u> die Web-App in ihrer Arbeit einsetzen?

Standardisierung der Versorgung

- Eines der Projektziele war es, die Versorgungsqualität an SPZ zu verbessern. Inwiefern wurde dies an Ihrem SPZ erreicht? Woran machen Sie das fest?
  - *<u>Vertiefen: Warum</u> ist dies für die Versorgungsqualität <u>wichtig</u>?*
  - *<u>Gelingen:</u> Welche Faktoren haben diese Qualitätsverbesserung unterstützt?*
  - *<u>Barrieren:</u> Was sind Barrieren für diese?*
- Inwiefern hat PART-CHILD dazu geführt, dass in Ihrem SPZ neue Standards in der Versorgung der Patienten eingeführt wurden?
  - *<u>Vertiefen:</u> Warum ist dieser Aspekt der Standardisierung wichtig?*
  - *<u>Gelingen</u>: Was waren die wichtigen Förderfaktoren?*
  - *<u>Barrieren</u>: Welche Barrieren gab es/gibt es?*
- Welche Rolle spielten die Inhalte der Mitarbeiter-Schulungen bei den *[beschriebenen Veränderungen]* hin zu einer höheren Versorgungsqualität bzw. Standardisierung?
- Inwiefern nützt hier die Verwendung der ICF-CY [→ Codieren]?
  - *<u>Gelingen:</u> Was machte die Anwendung der ICF-CY einfach?*
  - *<u>Barrieren:</u> Welche Barrieren gab es bei der Anwendung der ICF-CY?*

4. Bilanzierungsfragen (Wrapping up)

- Aus Ihrer bisherigen Erfahrung im PART-CHIILD-Projekt: Haben Sie für uns Anregungen, wie wir die PART-CHILD-Intervention in anderen SPZ bestmöglich einführen können?
  - *<u>Barrieren:</u>* Welche Barrieren könnte es bei der Einführung geben?

5. Nachgespräch (Feedback)

- Möchten Sie noch etwas zum Thema hinzufügen, über das wir nicht gesprochen haben?

- Wie ist es Ihnen während des Interviews ergangen?

Zum Schluss habe ich noch ein paar Fragen für unsere Statistik

Geschlecht Interviewpartner: ☐ m ☐ w

Fachkraftgruppe:

☐ Ärzte
☐ Psychologen
☐ Therapeuten
☐ Andere

Wie lange üben Sie diese Tätigkeit bereits aus? _________
Wie lange arbeiten Sie bereits in diesem SPZ? _________

Besonderheiten während des Gesprächs/ Anmerkungen/ Beobachtungen

Leitfaden für die telefonischen Interviews mit den SPZ-Leitungen

Interviewer: _______________
ID: ______________
SPZ_ID: ______________
Datum: ______________
Start: ______________ Ende: ___________

1. Einleitung

- Vorstellung der Interviewerin
- Erklärung zum Ablauf des Interviews, Erklärung zu spontanen Äußerungen und freier Assoziation.
- Hinweis auf Aufzeichnung

Im Folgenden geht es um Ihre Erfahrungen im Rahmen der PART-CHILD-Intervention. Mit Intervention meinen wir sowohl die Mitarbeiterschulung (Haben Sie an dieser teilgenommen?) das ICF-Add-In/die Web-App und die Begleitung im SPZ-Alltag (Modul IMPLEMENT). Mit der Intervention meinen wir aber nicht die Datenerhebung, also nicht die Fragebögen, die Sie austeilen und auch selbst ausfüllen mussten.

2. Eingangsfragen (Warm up)

- Wie kommt die Teilnahme am PART-CHILD-Projekt Ihrer Einschätzung nach in Ihrem Team an?
- Was war denn ausschlaggebend für Ihre Bewerbung um die Teilnahme am Projekt?

3. Hauptfragen

- Wenn Sie die vergangenen Monate Revue passieren lassen: Inwiefern hat sich die Teilnahme an dem Projekt für Ihr SPZ <u>gelohnt</u>?
- Was schätzen Sie: Wie <u>profitieren die Patienten und Eltern</u> an Ihrem SPZ davon, dass Ihr SPZ am Projekt teilgenommen hat?

Haltung der Leitung

- Welche Elemente aus der PART-CHILD-Intervention setzen Sie in Ihrem Arbeitsalltag ein?
    - *ICF-CY (z.B. Erfassung teilhaberelevanter Domänen nach Lakmoshibek, Codieren)?*
        - *Teilhabeorientierung & Patientenzentrierung: Therapieziele (Teilhabestatus/Teilhabepräferenz / Aktionsplan/Handlungsplan) werden zusammen mit Patienten nach bestimmten Kriterien festgelegt, Gespräche nach bestimmten Schemata geführt)*
    - *MI-Werkzeugkoffer?*
- Wie nutzen Sie derzeit ICF-CY?
    - *Warum ist es für Sie persönlich <u>wichtig</u>, ICF-CY anzuwenden?*
    - *<u>Gelingen</u>: Welche Faktoren sind bei der Einführung der ICF-CY förderlich?*
    - *<u>Barrieren</u>: Welche Barrieren gibt es bei der Nutzung der ICF-CY?*
    - *Wie wenden Ihre <u>KollegInnen</u> die ICF-CY an? Unterschiede zwischen Disziplinen?*
- Welche <u>Veränderungen</u> beobachten Sie bei Ihrer <u>eigenen Einstellung</u> zu Ihrer Tätigkeit?

Organisationale Veränderungen

- Was hat sich durch die Teilnahme am Projekt im Arbeitsalltag Ihres SPZ insgesamt verändert?
    - *Allgmein: Z.B. Abläufe, Arbeitsweise, mehr Zeit für den Patienten, Dokumentationsaufwand*
    - *Vertiefung: Wenn mehreres: Was war für Sie als Leitung die wichtigste Veränderung?*
    - *Warum hat sich das verändert?*
    - *Gelingen: Was war wichtig, damit diese Veränderung gelingen konnte?*
    - *Barrieren: Was waren Barrieren für diese Veränderung?*

- Was hat sich durch die PART-CHILD-Intervention im Team Ihres SPZ verändert?
    - *Welche Fachgruppen tun sich mit dieser Veränderung besonders leicht?*
        - *Warum denken Sie ist das so?*
    - *Welche Berufsgruppen tun sich besonders schwer mit der Veränderung?*
        - *Warum, denken Sie ist das so?*
        - *Wie könnte man diese Berufsgruppen in diesem Veränderungsprozess unterstützen?*
    - *Zusammenarbeit zwischen verschiedenen Disziplinen? Interdisziplinäre Verständigung?*
    - *Kommunikation mit anderen Abteilungen in der Klinik?*
- Wenn Sie an die Kommunikation in Ihrem SPZ denken: Welche Rolle spielt ICF dabei?
    - *(z.B. Teilhabeorientierung & Patientenzentrierung: Therapieziele (Teilhabestatus/Teilhabepräferenz / Aktionsplan/Handlungsplan) werden zusammen mit Patienten nach bestimmten Kriterien festgelegt, Gespräche nach bestimmten Schemata geführt)*
    - *Z.B. sind ICF-Themen Gegenstand von Routinebesprechungen?*
    - *Wird das Arbeiten nach ICF als ein Qualitätskriterium nach Außen kommuniziert?*

Fragen für Standorte, in welchen auch Teams der Frühförderung geschult werden

- Was hat sich durch die PART-CHILD-Intervention in der Zusammenarbeit zwischen dem SPZ und der Frühförderstelle verändert?
- Gab es generelle Unterschiede, wie diese beiden Einrichtungen/ Abteilungen die Inhalte der Teamschulungen in ihre Praxis umgesetzt haben?

- Welche strukturellen Veränderungen haben sich nach der Mitarbeiterschulung an Ihrem SPZ ergeben? / Was konkret tun Sie bereits, damit es weitergeht?
    - *Neue Arbeitsgruppe gebildet; MI-Multiplikatoren; Werden neue Mitarbeiter strukturiert in die Schulungsinhalte eingeführt?*
    - *Warum war es wichtig, diese Struktur einzuführen?*
    - *Wer war der Initiator der Veränderung (Team/ Leitung)?*

Nutzung von Add-In/Web-App

- In Ihrem SPZ wurde Ihnen die Web-App zum praktischen Arbeiten nach ICF-CY zur Verfügung gestellt. War die App eine sinnvolle Unterstützung bei der praktischen Umsetzung der Schulungsinhalte?
    - *Wie sollte sie in weiteren SPZ verwendet werden?*
        - *Flächendeckend einführen oder eher optionale Nutzung anbieten?*

Standardisierung der Versorgung

- Eines der Projektziele war es, die Versorgungsqualität an SPZ zu verbessern. Inwiefern wurde dies an Ihrem SPZ erreicht?
    - *Vertiefen: Woran machen Sie das fest?*
    - *Warum ist dies für die Versorgungsqualität wichtig?*
    - *Gelingen: Welche Faktoren haben diese Qualitätsverbesserung unterstützt?*
    - *Barrieren: Was sind Barrieren für diese?*
- Inwiefern hat PART-CHILD dazu geführt, dass in Ihrem SPZ neue Standards in der Versorgung der Patienten eingeführt wurden?
    - *Warum ist dieser Aspekt der Standardisierung wichtig?*
    - *Halten sich alle Fachkräfte an die neuen Standards?*
    - *Gelingen: Was waren die wichtigen Förderfaktoren?*
    - *Barrieren: Welche Barrieren gab es/gibt es?*
- Welche Rolle spielten die Inhalte der Mitarbeiter-Schulungen bei den *[beschriebenen Veränderungen]* hin zu einer höheren Versorgungsqualität bzw. Standardisierung der Versorgung?
- Inwiefern nützt hier die Verwendung der ICF-CY [→ Codieren]?
    - *Gelingen: Was machte die Anwendung der ICF-CY einfach?*
    - *Barrieren: Welche Barrieren gab es bei der Anwendung der ICF-CY?*

Kontextfaktoren

In der Evaluation versuchen wir Kontextfaktoren zu ermitteln, die für die Umsetzung der Inhalte der PART-CHILD-Intervention eine Rolle spielen.

- Gelingen: Welche Ressourcen bräuchte es, damit Sie in Ihrem SPZ möglichst viele Elemente der PART-CHILD-Intervention umsetzen können?
    - *Strukturell?*
    - *Personell?*
    - *Qualifizierungsmaßnahmen (Personal-/Organisationsentwicklung)*

- Barrieren: Welche *[weiteren, eher SPZ-bezogenen]* Barrieren gibt es Ihrer Einschätzung nach, die die Arbeit nach ICF-CY in Ihrem SPZ beeinflussen? Warum sind diese wichtig?

4. Bilanzierungsfragen (Wrapping up)

- Und wenn Sie die Datenerhebung kurz außer Acht lassen: Was sollte an der Intervention selbst verändert werden, damit sie in anderen SPZ ankommt?
- Aus Ihrer bisherigen Erfahrung im PART-CHIILD-Projekt: Haben Sie für uns Anregungen, wie wir die PART-CHILD-Intervention in anderen SPZ bestmöglich einführen können?
    - *Barrieren: Welche Barrieren könnte es bei der Einführung geben?*
- Sie haben uns berichtet, dass die PART-CHILD-Intervention Veränderungen in Ihrer Arbeits- und Denkweise mit sich brachte. Wenn Teilhabeorientierung und patientenzentrierte Arbeit mit ICF-CY zur gemeinsamen Perspektive für alle Einrichtungen werden soll, die mit Kindern mit chronischen Erkrankungen arbeiten:
    - Gelingen: Was bräuchte es dafür?
    - Barrieren: Mit welchen Barrieren sollte man rechnen?

5. Nachgespräch (Feedback)

- Möchten Sie noch etwas zum Thema hinzufügen, über das wir nicht gesprochen haben?
- Wie ist es Ihnen während des Interviews ergangen?

Geschlecht Interviewpartner: ☐ m ☐ w

Besonderheiten während des Gesprächs/ Anmerkungen/ Beobachtungen

Online Supplementary File S4: Completed SPIRIT Checklist

Section/item	Item No	Description
**Administrative information**		
Title	1	Descriptive title identifying the study design, population, interventions, and, if applicable, trial acronym (page 1)
Trial registration	2a	Trial identifier and registry name. If not yet registered, name of intended registry (page 4)
	2b	All items from the World Health Organization Trial Registration Data Set (not applicable)
Protocol version	3	Date and version identifier (not applicable)
Funding	4	Sources and types of financial, material, and other support (page 29)
Roles and responsibilities	5a	Names, affiliations, and roles of protocol contributors (pages 1 and 28)
	5b	Name and contact information for the trial sponsor (page 2)
	5c	Role of study sponsor and funders, if any, in study design; collection, management, analysis, and interpretation of data; writing of the report; and the decision to submit the report for publication, including whether they will have ultimate authority over any of these activities (page 29).
	5d	Composition, roles, and responsibilities of the coordinating centre, steering committee, endpoint adjudication committee, data management team, and other individuals or groups overseeing the trial, if applicable (see Item 21a for data monitoring committee) (not applicable)
**Introduction**		
Background and rationale	6a	Description of research question and justification for undertaking the trial, including summary of relevant studies (published and unpublished) examining benefits and harms for each intervention (pages 6 – 8)
	6b	Explanation for choice of comparators (not applicable)
Objectives	7	Specific objectives or hypotheses (page 9)
Trial design	8	Description of trial design including type of trial (eg, parallel group, crossover, factorial, single group), allocation ratio, and framework (eg, superiority, equivalence, noninferiority, exploratory) (page 9)

Methods: Participants, interventions, and outcomes

Study setting	9	Description of study settings (eg, community clinic, academic hospital) and list of countries where data will be collected. Reference to where list of study sites can be obtained (page 10)
Eligibility criteria	10	Inclusion and exclusion criteria for participants. If applicable, eligibility criteria for study centres and individuals who will perform the interventions (eg, surgeons, psychotherapists) (page 12 and 18)
Interventions	11a	Interventions for each group with sufficient detail to allow replication, including how and when they will be administered (pages 12 – 15)
	11b	Criteria for discontinuing or modifying allocated interventions for a given trial participant (eg, drug dose change in response to harms, participant request, or improving/worsening disease) (not applicable)
	11c	Strategies to improve adherence to intervention protocols, and any procedures for monitoring adherence (eg, drug tablet return, laboratory tests) (page 23)
	11d	Relevant concomitant care and interventions that are permitted or prohibited during the trial (not applicable)
Outcomes	12	Primary, secondary, and other outcomes, including the specific measurement variable (eg, systolic blood pressure), analysis metric (eg, change from baseline, final value, time to event), method of aggregation (eg, median, proportion), and time point for each outcome. Explanation of the clinical relevance of chosen efficacy and harm outcomes is strongly recommended (page 20)
Participant timeline	13	Time schedule of enrolment, interventions (including any run-ins and washouts), assessments, and visits for participants. A schematic diagram is highly recommended (see Figure) (pages 19 – 20)
Sample size	14	Estimated number of participants needed to achieve study objectives and how it was determined, including clinical and statistical assumptions supporting any sample size calculations (pages 17 – 18)
Recruitment	15	Strategies for achieving adequate participant enrolment to reach target sample size (pages 19 – 20)

Methods: Assignment of interventions (for controlled trials)

Allocation:

Sequence generation	16a	Method of generating the allocation sequence (eg, computer-generated random numbers), and list of any factors for stratification. To reduce predictability of a random sequence, details of any planned restriction (eg, blocking) should be provided in a separate document that is unavailable to those who enrol participants or assign interventions (page 17)

Allocation concealment mechanism	16b	Mechanism of implementing the allocation sequence (eg, central telephone; sequentially numbered, opaque, sealed envelopes), describing any steps to conceal the sequence until interventions are assigned (page 17)
Implementation	16c	Who will generate the allocation sequence, who will enrol participants, and who will assign participants to interventions (page 17)
Blinding (masking)	17a	Who will be blinded after assignment to interventions (eg, trial participants, care providers, outcome assessors, data analysts), and how (page 17)
	17b	If blinded, circumstances under which unblinding is permissible, and procedure for revealing a participant's allocated intervention during the trial (not applicable)
**Methods: Data collection, management, and analysis**		
Data collection methods	18a	Plans for assessment and collection of outcome, baseline, and other trial data, including any related processes to promote data quality (eg, duplicate measurements, training of assessors) and a description of study instruments (eg, questionnaires, laboratory tests) along with their reliability and validity, if known. Reference to where data collection forms can be found, if not in the protocol (pages 19 – 20)
	18b	Plans to promote participant retention and complete follow-up, including list of any outcome data to be collected for participants who discontinue or deviate from intervention protocols (not applicable)
Data management	19	Plans for data entry, coding, security, and storage, including any related processes to promote data quality (eg, double data entry; range checks for data values). Reference to where details of data management procedures can be found, if not in the protocol (page 20)
Statistical methods	20a	Statistical methods for analysing primary and secondary outcomes. Reference to where other details of the statistical analysis plan can be found, if not in the protocol (pages 20 – 21)
	20b	Methods for any additional analyses (eg, subgroup and adjusted analyses) (not applicable)
	20c	Definition of analysis population relating to protocol non-adherence (eg, as randomised analysis), and any statistical methods to handle missing data (eg, multiple imputation) (page 20)

Methods: Monitoring

Data monitoring	21a	Composition of data monitoring committee (DMC); summary of its role and reporting structure; statement of whether it is independent from the sponsor and competing interests; and reference to where further details about its charter can be found, if not in the protocol. Alternatively, an explanation of why a DMC is not needed (not applicable)
	21b	Description of any interim analyses and stopping guidelines, including who will have access to these interim results and make the final decision to terminate the trial (not applicable)
Harms	22	Plans for collecting, assessing, reporting, and managing solicited and spontaneously reported adverse events and other unintended effects of trial interventions or trial conduct (not applicable)
Auditing	23	Frequency and procedures for auditing trial conduct, if any, and whether the process will be independent from investigators and the sponsor (not applicable)

Ethics and dissemination

Research ethics approval	24	Plans for seeking research ethics committee/institutional review board (REC/IRB) approval (page 26)
Protocol amendments	25	Plans for communicating important protocol modifications (eg, changes to eligibility criteria, outcomes, analyses) to relevant parties (eg, investigators, REC/IRBs, trial participants, trial registries, journals, regulators) (not applicable)
Consent or assent	26a	Who will obtain informed consent or assent from potential trial participants or authorised surrogates, and how (see Item 32) (page 26)
	26b	Additional consent provisions for collection and use of participant data and biological specimens in ancillary studies, if applicable (not applicable)
Confidentiality	27	How personal information about potential and enrolled participants will be collected, shared, and maintained in order to protect confidentiality before, during, and after the trial (page 26)
Declaration of interests	28	Financial and other competing interests for principal investigators for the overall trial and each study site (page 29)
Access to data	29	Statement of who will have access to the final trial dataset, and disclosure of contractual agreements that limit such access for investigators (not applicable)

Ancillary and post-trial care	30	Provisions, if any, for ancillary and post-trial care, and for compensation to those who suffer harm from trial participation (not applicable)
Dissemination policy	31a	Plans for investigators and sponsor to communicate trial results to participants, healthcare professionals, the public, and other relevant groups (eg, via publication, reporting in results databases, or other data sharing arrangements), including any publication restrictions (page 26)
	31b	Authorship eligibility guidelines and any intended use of professional writers (not applicable)
	31c	Plans, if any, for granting public access to the full protocol, participant-level dataset, and statistical code (not applicable)
**Appendices**		
Informed consent materials	32	Model consent form and other related documentation given to participants and authorised surrogates (Online Supplementary File 5)
Biological specimens	33	Plans for collection, laboratory evaluation, and storage of biological specimens for genetic or molecular analysis in the current trial and for future use in ancillary studies, if applicable (not applicable)

Online Supplementary File S5: Consent forms

Medizinische Fakultät Mannheim
der Universität Heidelberg
Universitätsklinikum Mannheim

Mannheimer Institut für Public Health
Direktor: Prof. Dr. med. Joachim Fischer
Projektleitung: Prof. Dr. med. Freia De Bock
Vertretung: Dr. Tatiana Görig, Dr. med. univ. Michael Eichinger

Ludolf-Krehl-Str. 7-11, 68167 Mannheim
Telefon: +49 621 383-71834, Telefax: +49 621 383-71801
E-Mail: partchild@medma.uni-heidelberg.de

PART-CHILD
Verbesserung der Versorgungsqualität für Kinder mit chronischen Erkrankungen und Behinderungen durch die Stärkung von Partizipation und Teilhabe in Sozialpädiatrischen Zentren

Einwilligungserklärung der Eltern
zur Teilnahme an der Befragung
im Rahmen des wissenschaftlichen Projekts PART-CHILD

Liebe Eltern,

im Rahmen des Projekts PART-CHILD werden Daten über Sie und Ihr Kind erhoben. Die Weitergabe, Speicherung und Auswertung dieser projektbezogenen Daten erfolgt nach gesetzlichen Bestimmungen und setzt vor Teilnahme an dem Projekt folgende freiwillige Erklärung voraus:

Hiermit bestätige ich, dass ich über die Teilnahme an der wissenschaftlichen Befragung von meiner Fachkraft im SPZ umfassend und ausreichend informiert wurde. Ich und mein Kind hatten ausreichend Bedenkzeit, und alle unsere Fragen wurden im Vorhinein vollumfänglich beantwortet. Ich stimme auch im Sinne meines Kindes der Teilnahme an dieser Befragung freiwillig zu.

Ich erkläre mich einverstanden, dass im Rahmen der Studie erhobene Daten auf Fragebögen und/oder elektronischen Datenträgern aufgezeichnet und unter Nennung meines persönlichen Codes (pseudonymisiert) ausgewertet werden. Außerdem bin ich damit einverstanden, dass die Studiendaten in anonymisierter Form (d.h. ohne Namensnennung) für wissenschaftliche Darstellungen und Veröffentlichungen verwendet werden.

Mir ist bekannt,

(1) dass ich diese Einwilligung jederzeit ohne Angabe von Gründen und ohne Nachteile widerrufen kann (telefonisch unter: 0621-383-71834 oder per Email an: partchild@medma.uni-heidelberg.de),
(2) dass die Rechtmäßigkeit der bis zum Widerruf erfolgten Datenverarbeitung davon nicht berührt wird. In diesem Fall kann ich entscheiden, ob die von mir erhobenen Daten gelöscht werden sollen oder weiterhin für die Zwecke des wissenschaftlichen Projekts verwendet werden dürfen.

Ich möchte meine Daten ab Ende 2020 für weitere 10 Jahre für wissenschaftliche Auswertungen zur Verbesserung der Versorgungsqualität an SPZ zur Verfügung stellen.

Das Projektteam darf mir den Online-Fragebogen an folgende E-Mail-Adresse zuschicken:

____

Wenn ich unter der oben genannten E-Mail-Adresse nicht erreichbar bin, darf mir das Projektteam den Papierfragebogen an folgende Adresse senden:

____

☐ Ich stimme zu, dass mein Kind teilnehmen kann (wenn das Kind ≥ 7 Jahre alt ist).

☐ Ich stimme zu, dass ich nach Projektende gegebenenfalls ein weiteres Mal zum Ausfüllen eines kurzen Online-Fragebogens eingeladen werde. In diesem Fragebogen geht es ebenfalls um die Versorgungsqualität an Ihrem SPZ.

Die Originale der Elterninformation und der Einwilligungserklärung verbleiben bei mir. Den Erhalt der beiden Dokumente bestätige ich hiermit. Auf dem Forschungsdatenserver der Medizinischen Fakultät Mannheim der Universität Heidelberg wird lediglich eine elektronische Kopie der Einwilligungserklärung gespeichert. Den Inhalt der vorliegenden Einwilligungserklärung und der zugehörigen Informationsschrift habe ich verstanden.

Unterschreibt nur ein Erziehungsberechtigter, bestätigt dieser, dass er entweder das alleinige Sorgerecht innehat oder in Übereinstimmung mit dem anderen ebenfalls sorgeberechtigten Erziehungsberechtigten handelt.

Name des Erziehungsberechtigten in DRUCKBUCHSTABEN: ______________________________

Name des Erziehungsberechtigten in DRUCKBUCHSTABEN: ______________________________

Name des Kindes in DRUCKBUCHSTABEN: ______________________________

Ort, Datum: ______________________________

Unterschrift des Erziehungsberechtigten: ______________________________

Unterschrift des Erziehungsberechtigten: ______________________________

Aufklärende Person

Der Patient und seine Eltern wurden von mir im Rahmen eines Vorabgesprächs über das Ziel und den Ablauf des wissenschaftlichen Projekts aufgeklärt. Ein Exemplar der Informationsschrift und der Einwilligungserklärung habe ich dem Elternteil und dem Patienten ausgehändigt.

Name der aufklärenden Person in DRUCKBUCHSTABEN: ______________________________

Ort, Datum: ______________________________

Unterschrift der aufklärenden Person: ______________________________

Vergabe eines eigenen Codes für Ihre Angaben

Damit keine Rückschlüsse auf Ihre Person möglich sind, ist es notwendig, dass in den von Ihnen ausgefüllten Fragebögen statt Ihres Namens ein Code eingetragen wird. Dazu notieren Sie in den untenstehenden Kästchen wie folgt:

In die ersten zwei Kästchen notieren Sie die **zwei letzten** Buchstaben des **ersten Vornamens Ihres Kindes**.

In die nächsten zwei Kästchen notieren Sie die **zwei letzten** Buchstaben des **Nachnamens Ihres Kindes**.

In die folgenden zwei Kästchen notieren Sie den **Geburtstag der leiblichen Mutter** des Kindes.

In die letzten zwei Kästchen notieren Sie den **Geburtsmonat der leiblichen Mutter** des Kindes.

Beispiel: Das Kind heißt Pet**er** Michael Musterki**nd**. Seine leibliche Mutter hat am **09.04.** Geburtstag.

Der Code lautet daher:	E	R	N	D	0	9	0	4
☞**Bitte tragen Sie hier Ihren Code ein:**								

Liebe Eltern,

wir senden Ihnen eine E-Mail mit einem Link zu unserem Online-Fragebogen von unserer E-Mail-Adresse partchild@medma.uni-heidelberg.de.

☞ ***Bitte lassen Sie das SPZ-Team diese Einwilligungserklärung (alle 3 Seiten!) kopieren.***

Version für: **Kinder ≥ 10 Jahre**

Medizinische Fakultät Mannheim der Universität Heidelberg

Universitätsklinikum Mannheim

Mannheimer Institut für Public Health
Direktor: Prof. Dr. med. Joachim Fischer
Projektleitung: Prof. Dr. med. Freia De Bock
Vertretung: Dr. Tatiana Görig, Dr. med. univ. Michael Eichinger

Ludolf-Krehl-Str. 7-11, 68167 Mannheim
Telefon: +49 621 383-71834, Telefax: +49 621 383-71801
E-Mail: partchild@medma.uni-heidelberg.de

PART-CHILD
Verbesserung der Versorgungsqualität für Kinder mit chronischen Erkrankungen und Behinderungen durch die Stärkung von Partizipation und Teilhabe in Sozialpädiatrischen Zentren

Einwilligungserklärung der Kinder über 10 Jahre und Jugendlichen

zur Teilnahme im Rahmen des wissenschaftlichen Projekts PART-CHILD

Liebes Kind,
liebe Jugendliche, lieber Jugendlicher,

im Rahmen des Projekts PART-CHILD werden Daten über Dich erhoben. Die Weitergabe, Speicherung und Auswertung dieser projektbezogenen Daten erfolgt nach gesetzlichen Bestimmungen. Um teilzunehmen, musst du Folgendes freiwillig erklären:

Hiermit bestätige ich, dass ich über die Teilnahme an der Befragung von meiner Fachkraft im SPZ voll und ganz informiert wurde. Ich hatte ausreichend Bedenkzeit, und alle meine Fragen wurden im Vorhinein voll und ganz beantwortet. Ich stimme dieser Befragung freiwillig zu.

Außerdem bin ich damit einverstanden, dass meine Antworten von den Projektmitarbeitern gespeichert und ohne Namensnennung ausgewertet werden. Ich stimme auch zu, dass meine Antworten ohne Namensnennung in wissenschaftlichen Veröffentlichungen verwendet werden.

Ich weiß,

(1) dass meine Teilnahme vollkommen freiwillig ist. Ich weiß, dass ich meine Einwilligung jederzeit ohne Angabe von Gründen und ohne Nachteile zurückziehen kann. Meine Einwilligung kann ich zurückziehen telefonisch unter: 0621-383-71834 oder per E-Mail an: partchild@medma.uni-heidelberg.de.

(2) dass ich, wenn ich nicht mehr teilnehmen möchte, entscheiden kann, ob meine Antworten weiter verwendet werden dürfen.

Ich bin einverstanden, dass meine Antworten auch nach Projektabschluss Ende 2020 für weitere 10 Jahre für andere ähnliche wissenschaftliche Auswertungen verwendet werden.

Die Originale der Kinderinformation und der Einwilligungserklärung bleiben bei mir. Ich bestätige hiermit, dass ich beide Dokument erhalten habe. Auf dem Server der Universität Heidelberg wird lediglich eine elektronische Kopie der Einwilligungserklärung gespeichert.

Den Inhalt dieser Einwilligungserklärung und der zugehörigen Informationsschrift habe ich verstanden. Mit der beschriebenen Vorgehensweise bin ich einverstanden.

Name des Kindes/
des/der Jugendlichen in DRUCKBUCHSTABEN: ______________________________

Ort, Datum: ______________________________

Unterschrift des Kindes/des/der Jugendlichen: ______________________________

Aufklärende Person
Der Patient wurde von mir im Rahmen eines Vorabgesprächs über das Ziel und den Ablauf des wissenschaftlichen Projekts aufgeklärt. Ein Exemplar der Informationsschrift und der Einwilligungserklärung habe ich dem Patienten ausgehändigt.

Name der aufklärenden Person in DRUCKBUCHSTABEN: ______________________________

Ort, Datum: ______________________________

Unterschrift der aufklärenden Person: ______________________________

☞ ***Bitte die Mitarbeiter des SPZ diese 2 Seiten zu kopieren.***

Mannheimer Institut für Public Health
Direktor: Prof. Dr. med. Joachim Fischer
Projektleitung: Prof. Dr. med. Freia De Bock
Vertretung: Dr. Tatiana Görig, Dr. med. univ. Michael Eichinger

Ludolf-Krehl-Str. 7-11, 68167 Mannheim
Telefon: +49 621 383-71834, Telefax: +49 621 383-71801
E-Mail: partchild@medma.uni-heidelberg.de

PART-CHILD
Verbesserung der Versorgungsqualität für Kinder mit chronischen Erkrankungen und Behinderungen durch die Stärkung von Partizipation und Teilhabe in Sozialpädiatrischen Zentren

Einwilligungserklärung für Kinder unter 10 Jahren

Willst du an der Befragung im Projekt PART-CHILD teilnehmen?

Liebes Kind,

damit wir Dir ein paar Fragen stellen dürfen, musst Du uns für die nächsten Sätze Dein OK geben.

Ich bin einverstanden, dass ich an der Befragung teilnehme.
Ich bin einverstanden, dass meine Antworten ohne meinen Namen von den Forschern angeschaut werden.
Ich bin einverstanden, dass meine Antworten ohne meinen Namen von den Forschern gespeichert werden.

Alle meine Fragen wurden beantwortet.
Ich hatte genug Zeit, zu überlegen, ob ich mitmachen möchte.

Ich nehme ganz freiwillig teil.
Ich weiß, dass ich mich jederzeit anders entscheiden kann und nicht mehr an der Befragung teilnehmen muss.
Ich muss dafür keine Gründe nennen. Ich weiß, dass das nicht schlimm ist.
Wenn ich nicht mehr teilnehmen möchte, kann ich das

- Am Telefon: 0621-383-71834 sagen.
- In einer E-Mail: partchild@medma.uni-heidelberg.de schreiben.

Ich bin einverstanden, dass meine Antworten nach Projektende Ende 2020 weitere 10 Jahre auch für andere, ähnliche wissenschaftliche Auswertungen verwendet werden.

Ich habe verstanden, was hier und in der Informationsschrift geschrieben steht. Ich möchte mitmachen.

Schreibe hier Deinen Namen in DRUCKBUCHSTABEN auf: ______________________________

In welcher Stadt bist Du gerade? ______________________________

Welches Datum ist heute? ______________________________

Schreibe hier Deine Unterschrift hin: ______________________________

Aufklärende Person
Der Patient wurde von mir im Rahmen eines Vorabgesprächs über das Ziel und den Ablauf der Studie aufgeklärt.
Ein Exemplar der Informationsschrift und der Einwilligungserklärung habe ich dem Patienten ausgehändigt.

Name der aufklärenden Person in Druckbuchstaben: ______________________________

Ort, Datum: ______________________________

Unterschrift der aufklärenden Person: ______________________________

☞ ***Bitte die Mitarbeiter des SPZ diese Seite zu kopieren.***

Online Supplementary File S6: Framework for participation-centred care

	Elements	Explanation
	**Knowledge and attitudes concerning key concepts**	
1	Appreciation of participation-centredness	Commitment to (1) an extended view of a child’s health condition including the family’s perspectives and its implications for participation, (2) shared decision-making and (3) a guiding role of healthcare staff
2	Knowledge of participation-centred needs assessment	Basic knowledge about steps to conduct a participation-centred needs assessment under active involvement of children and their parents (e.g., joint clarification of concerns, participation restrictions and resources, formulation of participation goals)
3	Appreciation of interdisciplinarity	Appreciation of collaboration in interdisciplinary teams at eye level
4	Knowledge of the ICF-CY and other concepts related to participation	Knowledge about<br>● the ICF-CY and its components (body structures, body functions, activities, participation, personal factors and environment)<br>● the concept of functional health<br>● the biopsychosocial model of health<br>● Convention on the Rights of Persons with Disabilities
	**Skills and competencies**	
5	Clarification of concerns	Based on the professional experience of staff, identification and prioritisation (if needed) of concerns of children and their parents that made them seek care
6	Characterisation of daily life activities associated with the identified concerns	Joint description of daily life activities and their contexts associated with the children’s and parents’ concerns (identified in element 5) and their mapping to the nine ICF life domains (e.g., learning, communication, mobility, self-care)
7	Joint identification of areas in need of improved participation<br>and available resources	Joint identification and documentation of the life domains in need of improved participation and assessment of abilities in other life domains potentially useful for reaching improvement by patients, their parents and staff
8	Formulation of participation goals	Formulation of participation goals (based on the areas identified in element 7) emphasising the discrepancy between the goal and the status quo; the participation goals may be SMART (i.e., specific, measurable, achievable, relevant, time-bound), are attractive to the child and are under his/her control
9	Compilation of an ICF-CY profile	Identification of the most important aspects related to the participation goals (identified in element 8), mapping these to the ICF components and relating them to each other in preparation for the development of an action plan (element 11)
10	Identification of interventions	Joint identification of the most appropriate procedures, interventions or therapies to achieve the most important aspects of the participation goals (identified in element 8)

11	Development of an action plan	Agreement and documentation of specific next steps taken to achieve the child's participation goals by addressing the what, how, until when and by whom for each step
	**Cross-cutting skills**	
	Use of ICF-CY codes in routine documentation	Use of ICF-CY codes to support a concise yet comprehensive documentation of information identified in the context of other elements of the framework (e.g., when compiling an ICF-CY profile)
	Application of principles and methods of motivational interviewing	Application of key principles (e.g., develop discrepancy, support self-efficacy) and methods of motivational interviewing (e.g., asking open questions, rolling with resistance) when clarifying concerns, finding consensus among all participants involved, identifying participation goals, or developing action plans
	Delivery of care in the context of the wider care network	Consideration of the wider care network comprising professionals from different sectors and professions during the entire care process and involvement of all relevant stakeholders when developing and implementing action plans
